# Are Changes in Thigh Muscle Concentric Strength Associated With Changes in Leg Function After a Youth Sport-Related Knee Injury?

**DOI:** 10.1177/19417381251352524

**Published:** 2025-07-30

**Authors:** Justin M. Losciale, Christina Y. Le, Linda K. Truong, Garrett S. Bullock, Cameron J. Mitchell, Michael A. Hunt, Jackie L. Whittaker

**Affiliations:** † Department of Physical Therapy, Faculty of Medicine, University of British Columbia, Vancouver, British Columbia, Canada; ‡ Arthritis Research Canada, Vancouver, British Columbia, Canada; § Edwin S.H. Leong Centre for Healthy Aging, Faculty of Medicine, University of British Columbia, Vancouver, British Columbia, Canada; ‖ Informatics, Decision-Enhancement, and Analytic Sciences Center of Innovation, George E. Wahlen Department of Veterans Affairs Medical Center, Salt Lake City, Utah; ¶ Division of Epidemiology, Department Internal Medicine, University of Utah School of Medicine, Salt Lake City, Utah; # Carnegie School of Sport, Leeds Beckett University, Leeds, UK; ** Football Players Worldwide (FIFPRO), Hoofddorp, The Netherlands; †† Department of Orthopaedic Surgery and Rehabilitation, Wake Forest University School of Medicine, Winston-Salem, North Carolina; ‡‡ Department of Biostatistics and Data Science, Wake Forest University School of Medicine, Winston, Salem, North Carolina; §§ School of Kinesiology, Faculty of Education, University of British Columbia, Vancouver, British Columbia, Canada; ‖‖ Centre for Aging SMART, University of British Columbia, Vancouver, British Columbia, Canada

**Keywords:** ACL, change over time, hop testing, muscle performance

## Abstract

**Background:**

Assess the association between changes in injured leg knee extension and flexion strength (peak torque) and self-reported and performance-based measures of leg function after a variety of youth sport-related, time-loss knee injuries.

**Hypothesis:**

There will be a relationship between changes in knee muscle strength and changes in measures of leg function in youth after a sport-related knee injury.

**Study Design:**

Prospective cohort study.

**Level of Evidence:**

Level 2.

**Methods:**

This was a secondary analysis of the Alberta Youth Prevention of Early Osteoarthritis (PrEOA) Cohort study (Edmonton) that included youth (11-19 years old) who had experienced a medical attention, time-loss, sport-related knee injury in the previous 4 months. Injured leg knee extensor and flexor concentric peak torque (isokinetic; 90 deg/s), triple hop distance, modified Y-balance test (YBT), and Knee injury and Osteoarthritis Outcome Score Sport subscale (KOOS_sport_) were assessed at baseline (≤4 months postinjury) and 6 and 12 months later. Adjusted associations between 6- and 12-month change in strength and functional measures were assessed using multivariable regression (95% CI).

**Results:**

Based on data from 106 participants (16.2 ± 1.8 years old), a 1 Nm increase in knee extensor strength (6-12 months) was associated with a 0.9 cm (95% CI, –0.5, 2.3) increase in hop distance. Similarly, every 1 Nm increase in knee flexor strength (6-12 months) was associated with a 0.3 cm (95% CI, –1.1, 1.7) increase in hop distance. Across other models, a 1 Nm increase in extensor or flexor strength was associated with a 0- to 0.3-point increase in KOOS_sport_ score.

**Conclusion:**

There was minimal-to-no longitudinal relationship between changes in knee extensor or flexor strength and changes in triple hop or YBT performance, or self-reported function within the first year after a youth sport-related knee injury.

Persistent deficits in self-reported and performance-based (eg, hopping) measures of lower-limb related function are common after traumatic knee joint injuries in active youth.^1,11,22,23,33,53,68^ A core focus of rehabilitation guidelines is to restore these impairments to facilitate return to activity/sport,^32,34,35,40,67^ and potentially reduce any long-term consequences, such as post-traumatic osteoarthritis (OA).^67^

One of the most common explanations for lower-limb performance-based deficits after knee injury is the drastic loss (and slow recovery) of thigh muscle (ie, knee extensors and flexors) strength and power.^57^ A common hypothesis is that increasing knee muscle strength should lead to improvements in function. This conviction is based on cross-sectional clinical and biomechanical studies demonstrating associations between knee muscle strength (or strength asymmetry),^25,26,37^ and measures of lower-limb function (eg, triple hop distance and self-reported measures).^7,10,22,39,47,54^ For example, people with greater knee extensor strength asymmetry exhibit lower self-reported function,^10,24,70^ and stiffer landings, ie, lower knee flexion angles,^25,37^ and knee extensor moments,^27,37^ during squatting/jumping/running tasks. In contrast, greater knee extensor strength is associated with higher triple-hop distance.^7^

The hypothesis that knee muscle strength and function are related is not unique to knee injuries, and has been investigated in people living with hip and knee OA,^5,18,52^ and adolescents with patellofemoral pain.^19,20^ Unfortunately, we lack longitudinal studies that assess the relationship between changes in knee muscle strength and a corresponding change in clinically relevant measures of lower-limb function (eg, self-reported or performance based such as hopping), and also account for other factors that might influence function (eg, sex, leg dominance, body mass) after traumatic knee injury.^36^ A longitudinal study into how thigh muscle strength change is related to functional performance change may assist in more effective intervention planning for youth athletes.

This study assessed the association between changes in injured limb knee extension and flexion strength (ie, peak torque) and self-reported (Knee injury and Osteoarthritis Outcome Score function in sport and recreation sub-scale; KOOS_sport_) and performance-based (triple hop for distance and modified Y-Balance Test; YBT) measures of lower-limb function during the first year after a youth sport-related knee joint injury. We also explored whether this relationship differed by injury type, given our sample included a mix of knee injuries.

## Methods

### Study and Analysis Protocol

The analysis protocol was published a priori (see Appendix 1, online only).

### Ethics

This study was approved by the University of Alberta Health Research Ethics Board, Health Panel, Alberta, Canada (Ethics ID Pro00063773). Participants provided written consent and/or assent (when applicable) and completed the Physical Activity Readiness Questionnaire (PAR-Q, 2002) before testing.

### Study Design

This is a secondary analysis of a prospective cohort study that tracked outcomes at the time of injury (≤4 months), and semi-annually for 2 years. Only the baseline (≤4 months after injury), 6-, and 12-month follow-ups were used in this analysis,^36^ because this period coincides with a typical rehabilitation period^32^ and when the greatest improvements in outcomes are seen.^8,38,57^ This study is reported according to the Strengthening the Reporting of Observational Studies in Epidemiology (STROBE) Statement cohort studies checklist.^63^

### Participants and Recruitment

Active youth (11-19 years old) from Alberta, Canada who experienced a first-ever, unilateral, traumatic knee injury during sporting activities within the past 4 months were enrolled. Knee injury was defined as any clinically-diagnosed knee injury (ligament, meniscus, or other intra-articular tibiofemoral or patellofemoral injury) that occurred during sport or recreational activity participation, required medical consultation, and disrupted participation on ≥1 occasion in the previous 4 months.^68^ Injury type was categorized based on clinical examination and supported by diagnostic imaging and surgical reports, when available. Exclusion criteria included pregnancy, other time-loss injury within 4 months before baseline testing, arthritis diagnosis, or any condition preventing participation in functional tests (eg, neurological conditions). Recruitment began in December 2016 and concluded in September 2020.

### Procedures

Participants were assessed at baseline (≤4 months from injury) and 6 and 12 months later. At each assessment, participants completed an online survey (ie, participant characteristics and patient-reported outcomes) housed on REDCap Version 8.6.5, Vanderbilt University),^16^ and height, body mass, isokinetic knee extensor and flexor strength, and functional tasks (a hop test and a modified YBT) testing by physiotherapists and undergraduate students who underwent formal training. As reported in other research from this cohort,^38^ medical management and rehabilitation was not standardized to represent the natural history of changes after injury.

### Sample Size Justification

An a priori power calculation indicated our models have 82.4% power to detect the relationships of interest (Online Appendix 1).

### Exposure Variables

The exposure variables were injured leg concentric knee extensor and flexor isokinetic strength measured at the 6- and 12-month follow-ups. These variables were kept continuous and in their native unit (ie, not normalized) for primary analyses.^12,41^

Bilateral isokinetic, concentric knee extensor, and flexor peak torque (Nm) at 90 deg/s over a knee ROM from 0° (±2°) (full knee extension) to 90° (±2°) were collected using computerized dynamometry (BTE PrimusRS). Participants sat on the dynamometer with the hip at 90° of flexion, their trunk vertical, and with straps secured across the chest and thighs. Participants performed 1 practice trial, had 1 minute of rest, and then performed 3 maximal effort repetitions while receiving verbal encouragement. The uninjured limb was tested first.

### Outcomes

Outcomes included self-reported knee function in sport and recreation (KOOS_sport_, 0-100), average triple-hop distance (cm), and average modified YBT total composite reach distance (cm) measured at 6- and 12-month follow-ups.

#### Self-Reported Knee Function in Sport and Recreation

The KOOS_sport_ subscale consists of 5 items that ask participants to rate their perceived difficulty with squatting, running, jumping, twisting/pivoting, and kneeling.^9,49^ Individual items are scored on a Likert scale (0-4) and then transformed to a 0 (worst) to 100 (best) score. The KOOS is valid and reliable, and a recommended measure in this population.^9,67^

#### Lower-Limb Functional Performance

The triple-hop test estimates lower-limb functional performance.^15,46^ Participants performed a sequence of 3 consecutive forward hops, with the goal of covering as far a distance as possible, while landing and maintaining balance on the final hop. Participants completed 2 practice trials, and then 2 maximal effort test trials. The tests were performed without shoes on. The distance (from starting point to heel position on the final hop) was measured using a standard flexible tape measure affixed to the ground. The average distance (cm) across 2 trials was used for analysis. The triple hop is a reliable and recommended measure of functional performance after knee joint injuries that is commonly used in return-to-sport test batteries,^6,40,67^ and may predict future self-reported function.^64^

The modified YBT estimates lower-limb dynamic balance.^15,46^ Participants performed a modified YBT, with 3 measurement tapes affixed to the floor in a “Y” shape. Participants were instructed to stand barefoot on 1 leg at the intersection of the “Y” with their hands on their hips (uninjured limb was tested first). Their goal was to reach as far as possible in each of the 3 directions (ie, anterior, posterolateral, and posteromedial), holding the furthest point for 2 seconds, then returning to the center without losing balance. Participants performed 3 maximum effort trials after 1 practice trial per direction. A maximum of 2 extra repetitions per direction were allowed if the participant failed to maintain balance, lifted or moved their stance foot, touched the ground with the moving leg, or removed their hands from their hips. The maximum distance reached was recorded for each trial, then averaged across the 3 trials for each reach direction. The averages for each direction were summed together to indicate a total composite reach distance (cm).

### Confounder Selection

A directed acyclic graph (DAG) was created using www.daggity.net to depict the hypothesized cause-effect relationship between knee muscle strength (exposure) and lower-limb function (outcome) (Online Appendix 2).^58,59^ This DAG contents were informed by a previous study and research team expertise.^39^ Methods of measurement are reported in Online Appendix 3.

### Statistical Analysis

All analyses were performed in R.^48^ Participant characteristics, confounding factors, and other covariates were summarized as means and standard deviations, median (interquartile range [IQR]), or counts (%) as indicated. Missing data were assessed, and data were assumed to be missing at random (Online Appendix 4),^56,64^ and imputed using multivariate imputation with chained equations using the ‘mice’ package, with 100 imputed datasets.^21,56,62^ Imputation details are provided in Online Appendix 4.

Potential nonlinear relationships between continuous variables and each outcome were assessed using fractional polynomials.^2,50^ All relationships were linear, and no transformations were performed. Continuous variables were kept continuous.^41^

The association between the change in knee extensor or flexor strength and triple-hop or YBT outcomes was assessed using multivariable linear regression (95% CI). Models using the KOOS_sport_ score as the outcome were assessed using fractional regression (95% CI; generalized linear model with a quasibinomial distribution and logit link function), which preserved the bounded nature of the KOOS_sport_ (0-100). Model coefficients were converted from logits to interpretable beta-coefficients on the proportion (ie, percentage) scale using the “marginal effects” package.^3^ All analyses were performed in the imputed data, and pooled following Ruben’s Rules.^51^ Due to the amount of missing data in the baseline triple-hop measurement (76%), only the change from 6- to 12-month follow-up strength and triple-hop measures were analyzed. For all other outcomes, the baseline to 6- and 12-month change were also used.

Sample size prevented us from adjusting for all potential confounders. Using the DAG, we chose to adjust for sex, previous exposure, and outcome values (for triple hop, 6-month measurement was used; for all other outcomes, baseline measurement was used), baseline measurements of body mass, age, and treatment received. Triple-hop distance and YBT models were also adjusted for limb dominance and baseline height, given their influence on these tests. KOOS_sport_ models were also adjusted for family income and baseline sport exposure. Confounder coefficients were not interpreted as they were not the target of estimation.^66^ Cluster-robust standard errors (clustered on participant ID) were used to account for repeated measures.^43^

The estimate(s) of interest were the adjusted beta coefficients (95% CI) for knee extensor or flexor peak torque at timepoint of interest, which represented the relationship between a 1-Nm strength change and outcome change over the analyzed time period, independent of included covariates. Model assumptions (linearity, normality of residuals, homoscedasticity) were checked from 5 randomly selected imputations for each analysis. All assumptions were met.

### Exploratory Analysis

Given the variety of injury types in our sample, an exploratory analysis assessed whether the relationships of interest were modified by injury type (anterior cruciate ligament [ACL] ± concomitant injury vs non-ACL tear injury) by adding an exposure × injury type interaction term in the same models.

### Sensitivity Analyses

Sensitivity analyses were conducted to assess the consistency of the results.^17^ These included (1) using normalized (to body mass) strength (Nm/kg) as the exposure; (2) changing the covariates to baseline sport type, baseline competition level, and baseline outcome and exposure values; and (3) performing a complete case analysis to assess robustness to attrition and missing data biases.^56^

## Results

### Participants

Participant characteristics, including injury types, are provided in Table 1. Testing occurred a median (IQR) of 1.3 (1.2) months (baseline), 7.8 (1.3) months (6-month timepoint), and 13.7 (2.1) months (12-month timepoint) postinjury.

**Table 1.** Participant characteristics

Characteristic	Injured (n = 106)
Sex, n (%)	
Female	63 (59.4)
Male	43 (40.6)
Age at injury, y*^a^*	16.2 (1.8)
Injury type, n (%)	
ACL+	60 (56.6)
Non-ACL	46 (43.4)
Non-ACL knee injury types, n (%)	
Ligament	12 (26)
Meniscus	11 (24)
Patellofemoral dislocation	8 (17)
Other*^b^*	15 (33)
Injured limb, n (%)	
Dominant	41 (38.7)
Nondominant	65 (61.3)
Household income, $CAD*^a^*	128,877 (29,726)
Height, cm*^a^*	169.7 (9.1)
Body mass, kg*^a^*	68.8 (17.7)
Fat free mass, kg*^a^*	54.2 (12.1)
Weekly sport exposure, minutes/week*^a^*	459.4 (346.7)
Sport type, n (%)	
Collision	38 (35.9)
Field/court	58 (54.7)
Individual	10 (9.4)
Competition level, n (%)	
Recreational	6 (5.7)
School	17 (16.0)
Club	61 (57.6)
University	5 (4.7)
Provincial	8 (7.6)
National	6 (5.7)
Treatment received at baseline	
None	13 (12.3)
Physiotherapy only	83 (78.3)
Surgery and physiotherapy	10 (9.4)

Data provided from the original nonimputed dataset. ACL, anterior cruciate ligament; ACL+, ACL with or without concomitant injury; Non-ACL, injuries not involving ACL; CAD, Canadian dollars.

a Mean ± SD.

b Injuries such as cartilage trauma or fractures (pooled estimates from imputed data).

### Missing Data

Online Appendix 4 provides a summary of missing data. Missing exposure and outcome data occurred at all timepoints, ranging from 23% to 32% and 13% to 53% respectively. Missing covariate data ranged from 0% to 76%.

### Exposure and Outcome Values

Table 2 summarizes the average peak torque (Nm), normalized (body mass) peak torque (Nm/kg), limb symmetry, and outcome values at each timepoint.

**Table 2.** Exposure and outcome values in multiply imputed data

	Mean (95% CI)
Knee extensor peak torque	
Baseline	
Raw, Nm	88.3 (79.9, 96.7)
Normalized to BM, Nm/kg	1.3 (1.2, 1.4)
LSI	77.7 (70.3, 85.1)
6-months	
Raw, Nm	114.5 (105.1, 123.9)
Normalized to BM, Nm/kg	1.6 (1.5, 1.8)
LSI	88.7 (82.7, 94.7)
12-months	
Raw, Nm	120.9 (112.6, 129.2)
Normalized to BM, Nm/kg	1.7 (1.6, 1.9)
LSI	89.8 (84.6, 95.1)
Knee flexor peak torque	
Baseline	
Raw, Nm	65.6 (58.6, 72.5)
Normalized to BM, Nm/kg	1.0 (0.9, 1.1)
LSI	84.4 (74.8, 93.9)
6 months	
Raw, Nm	83.6 (76.1, 91.1)
Normalized to BM, Nm/kg	1.2 (1.1, 1.3)
LSI	92.1 (85.2, 99.1)
12 months	
Raw, Nm	89.0 (82.1, 95.9)
Normalized to BM, Nm/kg	1.3 (1.2, 1.4)
LSI	92.8 (86.0, 99.6)
Triple hop, cm	
Baseline	
Injured limb	221.2 (8.4, 434.0)
LSI	65.3 (1.9, 128.6)
6 months	
Injured limb	320.4 (292.3, 348.4)
LSI	91.8 (84.4, 99.2)
12 months	
Injured Limb	334.4 (307.4, 361.5)
LSI	94.6 (87.8, 101.4)
KOOS_Sport_ (0-100)	
Baseline	36.0 (31.2, 40.8)
6 months	55.6 (49.5, 61.7)
12 months	61.7 (54.9, 68.5)
YBT composite reach, cm	
Baseline	
Injured limb	232.5 (217.5, 247.5)
LSI	98.3 (91.9, 104.7)
6-months	
Injured limb	241.3 (230.9, 251.6)
LSI	98.9 (94.3, 103.5)
12-months	
Injured limb	252.8 (243.4, 262.3)
LSI	99.6 (95.5, 103.8)

Values represent mean (95% CI). LSI values represent (injured leg/uninjured leg) × 100.

BM, body mass; KOOS, knee injury and osteoarthritis outcome score; LSI, limb symmetry index; YBT, Y-balance test.

### Association Between Changes in Knee Muscle Concentric Strength and Function

The results from each analysis are reported in Table 3, and each subsequent full model and sensitivity analysis are reported in Online Appendix 5.

**Table 3.** Associations between peak torque change and knee-related function change over each timeperiod

Outcome	KOOS_sport_ (0-100) AME, % (95% CI)*^a^*			Triple-hop distance, cm Beta (95% CI)	YBT composite reach distance, cm Beta (95% CI)		
Timeperiod*^b^*	0-6	6-12	0-12	6-12	0-6	6-12	0-12
Knee extension peak torque, Nm*^c^*	0.2 (0.0, 0.4)	0.3 (0.1, 0.6)	0.3 (0.0, 0.6)	0.9 (–0.5, 2.3)	0.2 (–0.2, 0.6)	0.1 (–0.4, 0.5)	0.1 (–0.4, 0.5)
Knee flexion peak torque, Nm*^c^*	0.1 (–0.1, 0.4)	0.1 (–0.2, 0.4)	0.2 (–0.1, 0.5)	0.3 (–1.1, 1.7)	0.3 (–0.1, 0.8)	0.0 (–0.7, 0.7)	0.0 (–0.6, 0.6)

AME, average marginal effect; BM, body mass; KOOS, knee injury and osteoarthritis outcome score; YBT, Y-balance test.

a Beta coefficients were back transformed to the response scale (ie, % KOOS score from 0 to 100).

b 0, baseline assessment; 6, 6-month follow-up; 12, 12-month follow-up.

c Primary models performed in multiply imputed data and pooled following Ruben’s Rules. Models were adjusted for sex, baseline age, baseline BM (kg), baseline sport exposure, baseline family income, baseline treatment, baseline exposure and outcome (KOOS), and adjusted for sex, baseline age, limb dominance, baseline body mass (kg), baseline height (cm), baseline treatment, baseline exposure and outcome (triple-hop and YBT outcome).

### Exploratory Analysis

Exploratory analysis results are reported in Table 4.

**Table 4.** Contrast of exposure-outcome relationship between injury types

Outcome	KOOS_sport_ (0-100) AME (95% CI) *^a^*			Triple-hop distance, cm Beta (95% CI)	YBT composite reach distance, cm Beta (95% CI)		
Timeperiod*^b^*	0-6	6-12	0-12	6-12	0-6	6-12	0-12
Knee extension peak torque, Nm*^c^*	0.1 (–0.1, 0.4)	0.1 (–0.2, 0.4)	0.1 (–0.3, 0.4)	0.0 (–1.2, 1.2)	0.1 (–0.4, 0.6)	0.1 (–0.5, 0.7)	0.2 (–0.4, 0.8)
Knee flexion peak torque, Nm*^c^*	0.0 (–0.3, 0.3)	0.1 (–0.3, 0.5)	0.1 (–0.4, 0.6)	0.1 (–1.4, 1.6)	0.2 (–0.4, 0.7)	0.2 (–0.5, 1.0)	0.3 (–0.4, 1.0)

Contrasts represent the interaction term, with the Non-ACL group as the reference. ACL, anterior cruciate ligament; AME, average marginal effect; BM, body mass; KOOS, knee injury and osteoarthritis outcome score; Non-ACL, injuries not involving ACL; YBT, Y-balance test.

a Beta coefficients were back transformed to the response scale (ie, % KOOS score from 0 to 100).

b 0, baseline assessment; 6, 6-month follow-up; 12, 12-month follow-up.

c Primary models performed in multiply imputed data and pooled following Ruben’s Rules, models were adjusted for sex, baseline age, income, baseline treatment, baseline sport exposure, baseline body mass, baseline exposure and outcome (KOOS), Adjusted for sex, baseline age, limb dominance, baseline treatment, baseline BM (kg), baseline height, baseline exposure and outcome (triple-hop and YBT outcome).

### Sensitivity Analysis

Sensitivity analyses are reported in Appendix 5. The findings of the primary results did not change when considering different confounders. Complete case analyses yielded a smaller magnitude of triple-hop associations. Normalized peak torque (Nm/kg) produced inflated associations and 95% CI width.

## Discussion

There were no clinically relevant relationships between longitudinal changes in thigh muscle strength and changes in either triple hop distance, YBT, or KOOS_sport_ over an initial 12-month period after a youth sport-related knee injury. Collectively, these findings suggest that improvements in thigh strength (concentric) may have little impact on these specific (or similar) measures of leg function in youth after a knee injury.

### Knee Extensor and Flexor Concentric Strength and Hop Function

Although it is commonly hypothesized that increasing knee strength would improve measures of function, this has not been assessed in a longitudinal study. The estimated relationship found indicates that a greater relative change in knee extensor (or flexor) strength would need to occur to get a potentially detectable change in triple-hop distance. For instance, the average participant (in this sample) would need to experience a change in knee extensor strength of ~24 Nm (~25%) over the 6-month period to see an associated change in triple-hop distance above a reported minimum detectable change value (10% change, ~22 cm).^6^ The 95% CIs also suggest this association could be in both directions, with a potential loss of triple-hop distance (24 Nm → −12 cm, using the lower bound of 95% CI) or a larger gain (24 Nm → 55 cm, using upper bound of 95% CI). These findings suggest cautiously that there may be a small group of patients who experience an increase in hop distance from knee extensor (and/or flexor) strengthening focused exercises, but others will not. It is unknown whether there are any additional factors that may delineate these groups. These results are not cause-and-effect, as many confounding factors were not accounted for. In addition, the estimated association could also be an artifact due to natural recovery (in combination with rehabilitation) that typically occurs over the first year after knee injury.

The current study provides preliminary evidence that the mechanisms of hop performance increase may be driven by other relevant (and more potent) pathways than knee extensor and flexor strength gain.^4^ These other pathways could include neuromuscular control, hopping mechanics,^29,30^ confidence/fear,^45,69^ a different component of muscle performance (eg, eccentric strength or rate of torque development),^28,31^ or muscle performance at a different joint (eg, ankle).^28^ It is also plausible that the relationship between strength gain and hop distance improvement may depend on other factors (ie, an interaction effect),^61^ such as biomechanics change (eg, redistribution of joint work across hip, knee, and ankle).^30,37^ It is also plausible that thigh strength gain has a larger time-based relationship with other relevant outcomes, including knee extensor moments during running/jumping/landing,^27^ reinjury risk,^55^ and knee OA risk.^44,67^ These hypotheses require careful assessment in longitudinal studies.

### The Lack of a Relationship Between Strength and Self-Reported Function

In the current study, there was no clinically relevant relationship between thigh muscle strength change and changes in self-reported knee function (or dynamic balance). This finding is in line with past cross-sectional evidence suggesting small relationships between measures of muscle performance and self-reported function.^13,14,39,60^ These results also mirror recent research assessing whether the benefit of exercise-based interventions on self-reported function is mediated by knee extensor strength gain in people living with hip and knee OA.^52^ In a meta-analysis (n = 1407), increasing knee extensor strength mediated just 2% of the intervention’s effect on self-reported function.^52^ Collectively, past evidence and the present findings support the hypothesis that self-reported function improvement may come from movement-based interventions that are specific to the questions being asked, and minimally from increased thigh strength.^13,14,39,52,60^ However, it is still possible that thigh strength gain could mediate self-reported function improvement in a knee-injured population during a structured and experimentally controlled rehabilitation program, which was not provided in the current study. The current findings do not imply that strength or derivatives of strength values (eg, limb symmetry index) have no potential prognostic value for forecasting future self-reported function.^10^

### Clinical Implications

Addressing the patient’s current impairments is a mainstay of rehabilitation,^34^ with knee muscle weakness a prominent impairment after knee-joint injury.^57^ This study cautiously adds that there may be a small group of patients who gain additional benefit (ie, hop performance change) to the (already) recommended thigh strengthening interventions.^32,34,67^

### Research Implications

Given the small and variable relationship identified between thigh muscle strength and triple-hop distance, there are other pathways that may be more potent drivers of hop improvement. These include confidence/fear, neuromuscular control, or a different component of knee muscle performance (amongst others). Researchers with readily accessible longitudinal data may be well suited to explore these proposed relationships. In addition, the current results suggest that the absolute performance on a hopping task and the YBT and a measure of self-reported function may not be ideal primary or secondary outcome choices for exercise interventions focused on improving strength in people after knee injury.

### Strengths and Limitations

By following participants from the time of injury (baseline within 4 months) to 1 year thereafter, we were able to assess for clinically relevant longitudinal associations and control for multiple confounders. Handling missing data helped avoid selection bias^65^; however, fully observed data are preferable to multiple imputation. Different sensitivity analyses enhanced the internal validity of these results.

This study is limited by the use of only concentric, isokinetic assessment at 90 deg/s, which is only one relevant component of muscle performance after knee joint injury. Improvements in tasks such as the hop and YBT may be more related to eccentric strength or power.^28,31^ In addition, lower-limb function was restricted to the absolute performance on the triple hop and a modified YBT, which do not fully encompass all possible clinically relevant measures of lower-limb function or ways function can be quantified (eg, via biomechanics of the task). These results should be generalized only to young athletes during the first year of knee injury recovery. This study did not standardize rehabilitation, which could attenuate the relationship(s) of interest. It is plausible that a well-designed strengthening program that improves strength above and beyond “preinjury” strength values may have a greater impact on function measures. We purposefully adjusted for baseline values of confounders to avoid biases related to time-varying confounding that is affected by the exposure.^42^ Future research, with a larger sample, is necessary to account for this additional, but necessary, complexity to estimate this causal effect.^42^

## Conclusion

There was minimal-to-no longitudinal relationship between changes in knee extensor and flexor concentric strength and changes in the absolute performance on 2 common clinical measures of lower-limb function (hop and balance task) or self-reported function within the first year after a youth, sport-related knee joint injury.

## Supplemental Material

sj-pdf-1-sph-10.1177_19417381251352524 – Supplemental material for Are Changes in Thigh Muscle Concentric Strength Associated With Changes in Leg Function After a Youth Sport-Related Knee Injury? Supplemental material, sj-pdf-1-sph-10.1177_19417381251352524 for Are Changes in Thigh Muscle Concentric Strength Associated With Changes in Leg Function After a Youth Sport-Related Knee Injury? by Justin M. Losciale, Christina Y. Le, Linda K. Truong, Garrett S. Bullock, Cameron J. Mitchell, Michael A. Hunt and Jackie L. Whittaker in Sports Health
