# Supplementary material for: Are Changes in Thigh Muscle Concentric Strength Associated With Changes in Leg Function After a Youth Sport-Related Knee Injury?
Source: Sports Health. 2025 Jul 30;18(2):298–306. doi: 10.1177/19417381251352524 (PMC12310612; doi:10.1177/19417381251352524)
Supplement: sj-pdf-1-sph-10.1177_19417381251352524 – Supplemental material for Are Changes in Thigh Muscle Concentric Strength Associated With Changes in Leg Function After a Youth Sport-Related Knee Injury? [file sj-pdf-1-sph-10.1177_19417381251352524.pdf]

ONLINE APPENDICES

Appendix 1: A-priori protocol

Appendix 2: Directed Acyclic Graph

Appendix 3: Measurement Methods and Details

Appendix 4: Missing Data Assessment and Imputation Details

Appendix 5: Full models and sensitivity analysis results

APPENDIX 1: A-priori protocol

The purpose of this study was to assess the association between changes in injured limb knee extension and flexion peak torque and changes in self-reported (Knee injury and Osteoarthritis Outcome Score function in sport and recreation sub-scale; $KOOS_{sport}$) and performance-based (triple hop and Y-Balance Test) measures of knee function during the first year after a knee joint injury in young athletes.

METHODS

Study Design

This is a secondary analysis of a prospective cohort study that tracked various clinical outcomes semi-annually from the time of injury (≤4-months) to 24-months later. Only the baseline, 6- and 12-month follow ups were used in this analysis because they correspond to the typical periods of rehabilitation and allow implications to directly inform rehabilitation.[34]

Participants and Recruitment

Study selection criteria and participant recruitment have been previously published.[31, 33] Young athletes (11-19 years old) who experienced a primary (i.e., first-ever), traumatic, knee joint injury during sporting activities within the past four months were enrolled. Knee injury was defined as any knee injury (clinical diagnosis of a ligament, meniscus, or other intra-articular tibiofemoral or patellofemoral injury) that occurred during participation in a sport or recreational activity, required medical consultation, and disrupted sports participation on ≥1 occasion in the previous four months.[60] Injury type was categorized based on clinical examination and supported by diagnostic imaging and surgical reports when available. Exclusion criteria included pregnancy, other time-loss injury within four months prior to baseline testing, arthritis diagnosis, or any condition preventing participation in functional tests (e.g., neurological conditions). Recruitment began in December 2016 and concluded in September 2020.

Procedures

After informed consent/assent was obtained, participants were assessed at baseline (≤4 months from injury), 6- and 12-months after baseline. At each follow-up, participants completed a participant characteristics questionnaire (REDCap 8.6.5, Vanderbilt University, Tennessee, USA)[19] and attended an in-person testing session. In-person testing included measurements of height (to the nearest 0.1 cm without shoes on a stadiometer, Model 402KL, Pelstar, McCook, Illinois, USA), body mass, body composition, isokinetic knee extensor and flexor strength, a hop test and a modified Y-Balance test.[17, 42] Study personnel included physiotherapists and undergraduate students who underwent formal training.

Sample Size Justification

In total, 106 participants are available in this cohort. Past evidence was used to determine a plausible effect size across similar exposure and outcome relationships,[36] a multiple linear regression model with a minimum R^2 of 0.15,[36] Bonferroni adjusted alpha (0.025),[57] an expected regression coefficient of 1 (i.e., 1 Nm change → 1 unit change in outcome), primary exposure standard deviation of 40 (knee extension torque), outcome standard deviation of 125 (triple hop),[8, 30] and a maximum of 10 model parameters has 82.4% power to detect the relationship(s) of interest. Power analysis was performed using the 'pwrss' package in R (pwrss.f.regression function).

The chosen effect size (estimated coefficient of 1) is the smallest effect that may be worthwhile to estimate. This allows sufficient power to estimate a relation that size or greater.

Exposure Variables

The exposure variables are knee extensor and flexor peak concentric, isokinetic torque (Nm) on the injured limb measured at 6-months and 12-month follow-ups. These variables were treated as continuous variables in their native unit (i.e., not normalized) for primary analyses as the target of estimation is the association between strength and function, independent of other covariates.[14, 29, 45] A 6- and 12-month time frame coincides with the typical period of rehabilitation, and when the greatest impairment and subsequent improvement are expected.[53]

Bilateral concentric knee extensor and flexor torque at 90°/second over a knee ROM from 0° ±2° (full knee extension) to 90° ±2° were collected using computerized dynamometry (BTE PrimusRS, Hanover, Maryland, USA). Participants were seated on the dynamometer with the hip at approximately 90° of flexion and their trunk vertical, with straps secured across the chest and thighs. Participants performed one practice trial, were given a minute of rest, and then performed three maximal effort repetitions while receiving verbal encouragement. The uninjured limb was tested first.

Outcomes

The outcomes are self-reported knee function in sport and recreation ($KOOS_{sport}$, 0-100), average triple hop distance (cm), and average YBT total reach (cm) measured at 6- and 12-month follow-ups.

Self-reported knee function in sport and recreation

The $KOOS_{sport}$ subscale[11] consists of 5-items that ask participants to rate their perceived difficulty with tasks such as running, squatting, and twisting.[11, 46] Individual items are scored on a Likert scale (0-4) and then transformed to a 0 (worst) to 100 (best) scale. The KOOS is a valid, reliable[11] and recommended[59] PROM for this population.[11]

Lower limb functional performance

The triple hop test was collected to estimate lower limb functional performance. Participants performed a sequence of three consecutive forward hops, with the goal of covering as far a distance as possible, while landing and maintaining balance on the final hop. Participants completed two maximal effort test trials after two practice trials, with distance measured using a standard flexible tape measure affixed to the ground. The average of the two trials (cm) was used for analyses. The triple hop is reliable[6] and a recommended measure of functional performance after knee joint injuries.[6, 59]

Modified Y-Balance Test

The modified YBT estimates lower leg dynamic balance.[17] Participants performed a modified YBT, with 3 measurement tapes affixed to the floor in a “Y”-shape. Participants were instructed to stand barefoot on one leg at the intersection of the “Y” with their hands on their hips. Their goal was to reach as far as possible in each of the 3 directions (i.e., anterior, posterolateral, and posteromedial), holding the furthest point for 2 seconds, then returning to the centre without losing balance. Participants performed 3 maximum effort trials after 1 practice trial per direction and per leg. A maximum of 2 extra repetitions per direction were allowed if the participant failed to maintain balance, lifted or moved their stance foot, touched the ground with the moving leg, or removed their hands from their hips. The maximum distance reached was recorded for each trial, then averaged across the 3 trials for each direction. The averages for each direction were summed together to indicate a total composite reach distance (cm).

Confounder Selection

A directed acyclic graph (DAG) was developed to depict the hypothesized cause-effect relationship between knee muscle strength (exposure) and knee function (outcome) (FIGURE 1).[55] This DAG was informed by a previous study,[36] but was revised to incorporate the time based nature of the analysis.[37, 40] The DAG was created using the free website, www.daggity.net.[56]

Figure 1. Directed Acyclic Graph

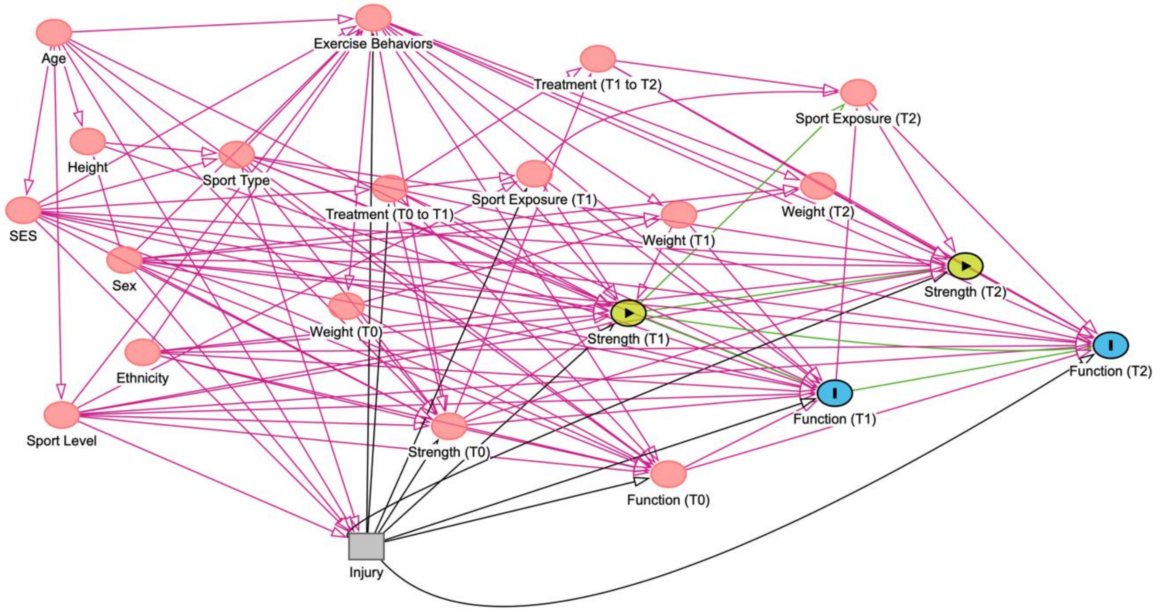

Based on the DAG, the confounders of interest include age (years; continuous),[50] participant sex (female, male; categorical)[10], limb dominance (dominant, non-dominant; categorical),[16] height (cm, continuous), body mass (kg; continuous), sport type (most frequently played; categorical),[39] competition level (recreational through elite; ordinal),[7] weekly sport exposure (minutes/week, continuous), average family income (continuous),[15] treatment (no treatment, PT-only, Surgery and PT, categorical), exercise behaviors (unmeasured), ethnicity (unmeasured), and the baseline values of the outcome and exposure (continuous). Continuous covariates were kept continuous in line with best practice guidance.[3, 45] Based on descriptive assessment of the data, height was considered to not be time varying, while body mass was considered time-varying.

Covariates of Interest

Age: Participant age (years) will be assessed using study specific questionnaire and used as a continuous variable in analyses as per statistical recommendations.[2, 3]

Sex: Participant sex (female, male, other)[10] will be assessed at baseline using study specific questionnaire and used as a categorical variable in analyses. Absolute strength and function can be different between sexes due naturally occurring differences in fat free mass.[41]

Height: height will be measured without shoes (to the nearest 0.1 cm) on a stadiometer, Model 402KL, Pelstar, McCook, Illinois, USA. Height is a strong prognostic factor for hop and YBT reach distance.[17]

Limb Dominance: Limb dominance (dominant vs. non-dominant) will be determined at baseline by asking participants which limb they would use to kick a ball as far as possible.[16] The dominant lower extremity is more likely to be injured.[16]

Body Mass: Body mass (kg) will be measured using a bioelectrical impedance analysis (BIA) unit Tanita Body Composition Analyzer MC-780U. Participants stand barefoot on the footplate and body weight will be estimated via the internal strain gauge load cell (±0.2 kg). The device will be calibrated prior to each scan (according to the manufacturer's protocol).

Sport Type: Sport type will be determined at baseline using study specific questionnaire to ascertain which sport the participant plays most frequently throughout the year. This will be treated as a categorical variable and assessed for sufficient overlap between participants prior to analysis. Specific sports raise the risk for knee injuries and require higher demand on the knee.

Competition Level: Competition will be determined at baseline using study specific questionnaire to ascertain at what level the participant was competing at during their primary sport. This will be treated as an ordinal variable (recreational through elite) and will be assessed for sufficient overlap between participants prior to analysis. Individuals at higher competition levels generally have higher risk for knee injuries but are also likely to participate in more formal programs to build muscle function.[7]

Weekly Sport Exposure: Weekly athletic exposure volume will be calculated for the primary sport using a study specific questionnaire. Participants will provide the number of sessions and minutes/session they participate in their main sport. The volume will be the product of minutes*sessions.

Socioeconomic Status: Since socioeconomic status (SES, yearly income) was not directly measured.[15] Participants' postal codes was used as a proxy measure for SES by imputing the median income value for that postal code for families with children based on values reported in National level Statistics (i.e., Statistics Canada) (https://www12.statcan.gc.ca/census-recensement/2016/dp-pd/prof/details/page.cfm?Lang=E&Geo1=FSA&Code1=K1V&Geo2=PR&Code2=01&SearchText=K1V1T9&SearchType=Begins&SearchPR=01&B1=Income&TABID=2&type=0).

Treatment: The treatment received for the index knee injury was recorded using a study specific questionnaire. Participants were able to report the types of treatments received from an exhaustive list. This variable will be assessed for sufficient overlap and categories will be collapsed where indicated.

Statistical Analysis
All analyses will be performed in R.[44]

Demographic characteristics, confounding factors, and other covariates were summarized as means ± SD, median (range), or counts (%) as indicated.

Missing data will be assessed, and data over 5% missing, identified as missing at random or missing completely at random will be imputed using multivariable imputation with chained equations using the 'mice'[9] package in R. Based on preliminary assessments of missing data, 100 imputed datasets will be created.[24, 52] All variables included in the imputation will be reported.

All continuous exposures and covariates will be assessed for nonlinear relationships with the outcomes via fractional polynomials (FPs).[3, 47] This will be performed within each imputed dataset, with the most used transformation (if any) carried forward into the outcome analysis. If any relationship is identified as nonlinear, an additional step will be performed to identify the best nonlinear transformation between FPs, log transformation, exponents, and restricted cubic splines (via the 'rms' package).[18] Model Akaike Information Criterion (AIC) values will be used to compare these transformations, with the transformation that generates the lowest AIC being selected for outcome modeling.

The association between each strength exposure and triple hop and YBT function outcome will be assessed using multivariable linear regression (95%CI). The association between each strength exposure and KOOS subscales will be assessed using fractional regression (95%CI). Fractional regression preserves the bounded nature of the outcome (0 to 100). This will be performed using a generalized linear model with a quasibinomial distribution and a logit link function. The beta coefficients will be converted from logits to interpretable beta-coefficients on the proportion (i.e., %) scale using the 'marginal effects' package.[4]

A model will be built for each time period, resulting in 2 models per outcome-knee muscle group exposure. For example, the association between strength and function from baseline to 6-months will be model 1, and the association between strength and function from baseline to 12-months will be model 2. This was selected because it allows for better identification of relationships with limited number of repeated measures.

Since this study is addressing these questions under a cause-effect framework, the estimate(s) of interest are the adjusted beta coefficient (95%CI) for each peak torque term at either the 6 or 12-month time point (depending on the analysis). A change

variable was not used because it does not align with the underlying analysis paradigm[54] and is computationally identical to the post value adjusted for the baseline value.

All models will be adjusted for the baseline outcome value, the baseline exposure value, age, sex, baseline body mass, and baseline treatment received since this was not standardized in this study. Since the outcomes defer in that two are performance based and one is self-reported, different additional covariates were added to the analyses. Specifically, models with triple hop and YBT as the outcome will also be adjusted for baseline height and limb dominance since there are strong conceptual basis that those who are taller may jump further (or reach further) and those who are hopping/balancing on their dominant leg may have differential performance. For the $KOOS_{sport}$ models, the additional confounders of baseline family income and baseline sport exposure will be added. Since this is a self-reported measure that relies on individual's perceptions, these likely are anchored against what they were previously able to do (sport exposure) and those with higher income may be more likely to engage in a more sport-based activities, all which may impact how they currently perceive their function.

Confounder coefficients will not be interpreted as they are not the target of estimation.[58] To account for repeated measures, cluster robust standard errors, clustered by participant, will be calaculated.[38]

Exploratory Analysis
An exploratory analysis will be performed to explore if the relationship(s) of interest differ by the type of injury experienced (ACL with/without concomitant injury vs. non-ACL tear injury). Using the primary models, an exposure^{x}injury type interaction term will be included. This is necessary due to the heterogenous sample of injury types. This analysis will also provide novel information regarding any unique differences that should be factored into rehabilitation.

Sensitivity analyses

Sensitivity analyses will be conducted to understand the consistency of the results against our analytical decisions.[20] These included 1) using normalized strength change (to body mass) as the exposure, 2) changing the adjusted confounders to baseline sport type, baseline competition level, and baseline outcome and exposure values, 3) performing a complete case analysis to assess our robustness to attrition and missing data biases.[52]

APPENDIX 2: Directed Acyclic Graph

Confounding factors included in the analysis were determined a-priori based on the below directed acyclic graphs (DAG) depicting the cause-effect relationship between knee strength (exposure) and leg function (outcome) (**Figure 1**).[8, 11, 12] The DAG was created using a search of the existing literature, expert knowledge, discussion amongst the research team, and the results of a previous study[30] showing the potential relationships between knee strength and function in a heterogenous sample of individuals after knee injury. Confounding factors were identified visually[13] following DAG graph theory[8, 11, 12] as being a factor that creates an open backdoor pathway from the exposure of interest to the outcome. This method also ensures we avoid collider-stratification bias.[10, 11] The DAGs were created using www.daggity.net.[15]

Due to sample size and measurement limitations, not all factors could be included in the analysis. The variables selected were based on conceptual plausibility.

Figure 1. Directed Acyclic Graph

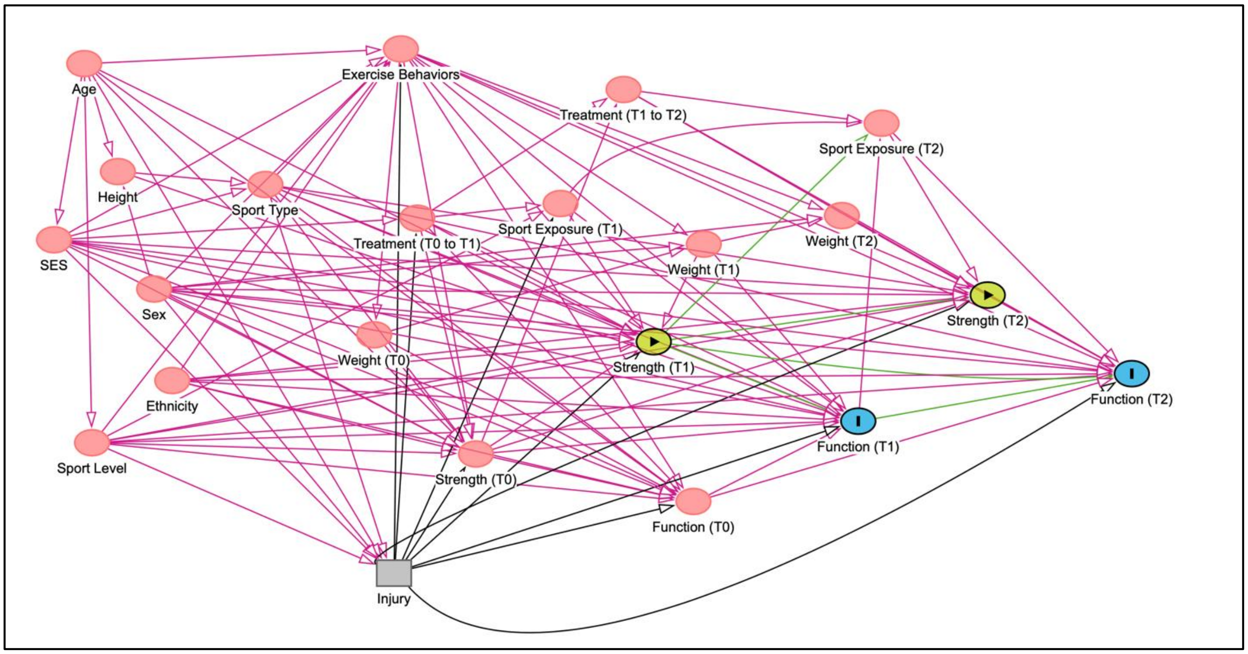

APPENDIX 3: Measurement Methods and Details

Age: Participant age (years) were assessed using study specific questionnaire and used as a continuous variable in analyses as per statistical recommendations.[2, 3]

Sex: Participant sex (female, male)[10] were assessed at baseline using study specific questionnaire and used as a categorical variable in analyses. Absolute strength and function can be different between sexes due naturally occurring differences in fat free mass.[41]

Height: Height was measured without shoes (to the nearest 0.1 cm) on a stadiometer, Model 402KL, Pelstar, McCook, Illinois, USA. Height is a strong prognostic factor for hop and YBT reach distance.[17]

Limb Dominance: Limb dominance (dominant vs. non-dominant) was determined at baseline by asking participants which limb they would use to kick a ball as far as possible.[16] The dominant lower extremity is more likely to be injured.[16]

Body Mass: Body mass (kg) was measured using a bioelectrical impedance analysis (BIA) unit Tanita Body Composition Analyzer MC-780U. Participants stand barefoot on the footplate and body weight will be estimated via the internal strain gauge load cell (±0.2 kg). The device will be calibrated prior to each scan (according to the manufacturer's protocol).

Sport Type: Sport type was determined at baseline using study specific questionnaire to ascertain which sport the participant plays most frequently throughout the year. This was examined for sufficient overlap and categories were collapsed into 'individual', 'field/court' or 'collision' sports. Individual sports included items such as swimming, figure skating, and dance/cheer/gymnastics. Field/court sports included soccer, basketball, and volleyball etc. Collision sports included football, rugby, wrestling, and hockey. Specific sports raise the risk for knee injuries and require higher demand on the knee.

Competition Level: Competition was determined at baseline using study specific questionnaire to ascertain at what level the participant was competing at during their primary sport. This was treated as an ordinal variable (recreational through elite) and categories were collapsed into recreational (self-reported), competitive (playing for a club or school team), and elite (playing at a university, provincal, or national level). Individuals at higher competition levels generally have higher risk for knee injuries but are also likely to participate in more formal programs to build muscle function.[7]

Weekly Sport Exposure: Weekly athletic exposure volume was calculated for the primary sport using a study specific questionnaire. Participants provided the number of sessions and minutes/session they participated in their main sport. The volume was the product of minutes*sessions.

Socioeconomic Status: Since socioeconomic status (yearly income) was not directly measured,[15] participants' postal codes were used as a proxy measure by imputing the median income value for that postal code for families with children based on values reported in National level Statistics (i.e., Statistics Canada) (https://www12.statcan.gc.ca/census-recensement/2016/dp-pd/prof/details/page.cfm?Lang=E&Geo1=FSA&Code1=K1V&Geo2=PR&Code2=01&SearchText=K1V1T9&SearchType=Begins&SearchPR=01&B1=Income&TABID=2&type=0).

Treatment: The treatment received for the index knee injury was recorded using a study specific questionnaire. Participants were able to report the types of treatments received from an exhaustive list. This variable was collapsed based on broad items into the categories 'no treatment', 'physiotherapy only' and 'surgery and physiotherapy'.

Appendix 4: Missing Data Assessment and Imputation Details

Comparison of those with and without missing data on baseline covariates, exposures, and additional outcomes are reported for each exposure and outcome (**Tables 1-14 below**). Missing data patterns were different at each time point. Missing data appears to be missing at random (i.e., data dependent),[32] with factors related to the injury and treatment, participant characteristics, and related measures of symptoms, function and quality-of-life predictive of missingness at varying time points.

Given missing at random mechanism, multiple imputation using chained equations was used to impute the missing values and avoid selection bias (i.e., collider bias).[8, 16, 18, 26] Prior to multiple imputation, data were pre-processed to remove/correct data entry errors and impossible values. When body characteristic variables such as height, weight or fat free mass were missing, data were interpolated if the missing value was between two observed values, (e.g., baseline- and 12-month are observed, and 6-month is missing). Random error was added using the jitter function in R. If interpolation was not deemed viable, the value was imputed in the multiple imputation process. When impossible values or data entry errors were found, these values were deleted and treated as a missing value.

Multiple Imputation Specifications and Models

Multiple imputation was performed in R using the mice package.[30] 100 imputed data sets were created, with 5-iterations each. Variables included in the imputation models included all variables in the outcome model, exploratory model, the past and future values of the variable being imputed, and any auxiliary variable with a minimum correlation of 0.60 and at least 60% observed data based on the missing data assessment (see below). The opposite limb value of each variable being imputed was also included (e.g., injured limb triple hop was used to impute the uninjured limb triple hop).

Data were imputed in wide format (1 row per participant). Continuous variables were imputed using linear regression ("norm" model) or predictive mean matching (for bounded measures), binary variables were imputed with predictive mean matching.

Derived or composite variables (e.g., normalized peak torque) were passively imputed by imputing each component of the ratio and then combining them passively to maintain the appropriate relationship. Feedback loops between passively imputed variables and their component parts (i.e., normalized strength and weight) were addressed by not allowing normalized strength (or the derived variable) to impute it's component parts.[29]

Table 1. Missing Data Assessment for the Triple Hop at Baseline

Variable	Not Missing (n=26)	Missing (N=80)
Knee Extension Torque (baseline)	95.3 (33.0)	88.4 (38.2)
Knee Extension Torque (6-month)	101.1 (29.2)	117.1 (32.2)
Knee Extension Torque (12-month)	119.7 (23.3)	123.4 (32.4)
Knee Flexion Torque (baseline)	65.5 (23.7)	64.9 (27.4)
Knee Flexion Torque (6-month)	75.2 (25.8)	85.1 (28.1)
Knee Flexion Torque (12-month)	86.7 (18.8)	86.3 (24.8)
KOOS Sport (baseline)	55.6 (22.2)	29.5 (20.3)
KOOS Sport (6-month)	77.2 (22.5)	49.6 (24.5)
KOOS Sport (12-month)	80.1 (18.3)	57.0 (27.4)
KOOS QoL (baseline)	73.5 (20.3)	45.3 (28.8)
KOOS QoL (6-month)	84.3 (18.3)	75.8 (22.3)
KOOS QoL (12-month)	91.4 (11.7)	80.8 (22.4)
KOOS Symptoms (baseline)	79.8 (14.6)	59.0 (16.8)
KOOS Symptoms (6-month)	87.9 (11.3)	80.3 (12.8)
KOOS Symptoms (12-month)	86.6 (11.1)	80.8 (16.2)
YBT Composite Reach (baseline)	236.1 (19.2)	231.6 (25.4)
YBT Composite Reach (6-month)	238.3 (24.6)	242.3 (28.4)
YBT Composite Reach (12-month)	253.4 (19.7)	254.0 (24.5)
Triple Hop (6-month)	349.5 (67.8)	332.8 (79.5)
Triple Hop (12-month)	375.6 (80.4)	334.4 (61.2)
Age (baseline)	15.6 (1.8)	16.6 (1.8)
Height (baseline)	166.2 (8.8)	170.8 (9.0)
Weight (baseline)	62.0 (13.5)	71.0 (18.4)
Sex		
F	17	46
M	9	34
Injury Type		
ACL	5	55
Non-ACL	21	25
Family Income	124,150.5 (32789.3)	130,413.1 (28714.6)
Weekly Sport Exposure	429.8 (262.0)	470.1 (373.7)
Limb Dominance of Injured Leg		
Dominant	16	49
Non-Dominant	10	31
Treatment (baseline)		
Surgery and PT	0	10
Competition Level (baseline)		
Recreational	2	4
Competitive	20	58
Elite	4	15
Sport Type (baseline)		
Collision	9	29
Field/Court	15	43
Individual	2	8

ACL (anterior cruciate ligament), F (female), KOOS (Knee injury and Osteoarthritis Outcome Score), M (male), PT (physiotherapy), QoL (quality of life), YBT (Y-Balance Test)

Table 2. Missing Data Assessment for the Triple Hop at 6-months

Variable	Not Missing (n=50)	Missing (N=56)
Knee Extension Torque (baseline)	90.4 (37.7)	90.4 (36.0)
Knee Extension Torque (6-month)	118.9 (35.4)	104.2 (24.4)
Knee Extension Torque (12-month)	123.3 (28.9)	121.5 (31.8)
Knee Flexion Torque (baseline)	67.0 (29.8)	63.1 (22.0)
Knee Flexion Torque (6-month)	87.4 (30.0)	76.5 (23.3)
Knee Flexion Torque (12-month)	87.8 (23.8)	85.2 (23.0)
KOOS Sport (baseline)	44.5 (24.0)	28.4 (20.7)
KOOS Sport (6-month)	63.9 (27.3)	48.0 (23.6)
KOOS Sport (12-month)	68.2 (28.9)	56.7 (24.4)
KOOS QoL (baseline)	62.3 (24.0)	43.4 (31.2)
KOOS QoL (6-month)	85.4 (15.1)	69.4 (24.8)
KOOS QoL (12-month)	86.2 (19.7)	80.5 (21.7)
KOOS Symptoms (baseline)	70.5 (17.4)	58.6 (17.8)
KOOS Symptoms (6-month)	84.3 (11.4)	79.9 (14.1)
KOOS Symptoms (12-month)	82.7 (14.4)	81.6 (16.3)
YBT Composite Reach (baseline)	234.3 (22.6)	231.8 (24.7)
YBT Composite Reach (6-month)	247.1 (26.6)	232.7 (26.7)
YBT Composite Reach (12-month)	257.2 (22.3)	250.4 (24.0)
Triple Hop (baseline)	329.3 (62.5)	342.4 (88.2)
Triple Hop (12-month)	353.9 (68.4)	339.5 (75.0)
Age (baseline)	16.1 (2.0)	16.5 (1.6)
Height (baseline)	169.7 (9.0)	169.7 (9.3)
Weight (baseline)	65.6 (14.9)	71.7 (19.5)
Sex		
F	29	34
M	21	22
Injury Type		
ACL	17	43
Non-ACL	33	13
Family Income	131,417.8 (30231.6)	126,608.4 (29352.4)
Weekly Sport Exposure	475.8 (419.2)	443.1 (258.3)
Limb Dominance of Injured Leg		
Dominant	30	35
Non-Dominant	20	21
Treatment (baseline)		
Surgery and PT	1	9
Competition Level (baseline)		
Recreational	4	2
Competitive	33	45
Elite	12	7
Sport Type (baseline)		
Collision	20	18
Field/Court	25	33
Individual	5	5

ACL (anterior cruciate ligament), F (female), KOOS (Knee injury and Osteoarthritis Outcome Score), M (male), PT (physiotherapy), QoL (quality of life), YBT (Y-Balance Test)

Table 3. Missing Data Assessment for the Triple Hop at 12-months

Variable	Not Missing (n=52)	Missing (N=54)
Knee Extension Torque (baseline)	92.2 (36.4)	88.3 (37.3)
Knee Extension Torque (6-month)	115.4 (35.8)	109.9 (26.8)
Knee Extension Torque (12-month)	128.2 (31.1)	110.3 (24.7)
Knee Flexion Torque (baseline)	67.0 (27.4)	62.8 (24.9)
Knee Flexion Torque (6-month)	85.6 (30.2)	79.0 (24.2)
Knee Flexion Torque (12-month)	88.9 (24.4)	81.9 (20.6)
KOOS Sport (baseline)	40.6 (23.4)	31.6 (23.1)
KOOS Sport (6-month)	60.9 (27.3)	50.9 (25.3)
KOOS Sport (12-month)	71.4 (22.4)	49.1 (28.7)
KOOS QoL (baseline)	58.5 (27.2)	46.3 (30.6)
KOOS QoL (6-month)	79.9 (20.1)	75.5 (23.4)
KOOS QoL (12-month)	89.9 (12.9)	73.5 (26.2)
KOOS Symptoms (baseline)	68.1 (17.5)	60.4 (18.8)
KOOS Symptoms (6-month)	82.6 (12.9)	81.8 (13.0)
KOOS Symptoms (12-month)	86.0 (11.1)	76.4 (18.7)
YBT Composite Reach (baseline)	235.5 (22.7)	230.1 (24.4)
YBT Composite Reach (6-month)	242.8 (28.6)	239.5 (26.1)
YBT Composite Reach (12-month)	253.2 (22.5)	255.2 (25.1)
Triple Hop (baseline)	321.2 (69.2)	359.0 (63.8)
Triple Hop (6-months)	337.9 (70.0)	341.6 (86.1)
Age (baseline)	16.2 (1.9)	16.4 (1.8)
Height (baseline)	169.5 (10.0)	169.9 (8.3)
Weight (baseline)	66.8 (18.2)	70.7 (17.1)
Sex		
F	30	33
M	22	21
Injury Type		
ACL	24	36
Non-ACL	28	18
Family Income	126,696.0 (30,105.9)	130,977.2 (29,483.1)
Weekly Sport Exposure	427.1 (255.4)	491.7 (419.1)
Limb Dominance of Injured Leg		
Dominant	29	36
Non-Dominant	23	18
Treatment (baseline)		
Surgery and PT	5	5
Competition Level (baseline)		
Recreational	3	3
Competitive	36	42
Elite	11	8
Sport Type (baseline)		
Collision	17	21
Field/Court	28	30
Individual	7	3

ACL (anterior cruciate ligament), F (female), KOOS (Knee injury and Osteoarthritis Outcome Score), M (male), PT (physiotherapy), QoL (quality of life), YBT (Y-Balance Test)

Missing Data Assessment for the KOOS Sport at Baseline
Not performed, only 2 missing values

Table 4. Missing Data Assessment for the KOOS Sport at 6-months

Variable	Not Missing (n=92)	Missing (N=14)
Knee Extension Torque (baseline)	88.9 (36.6)	107.8 (35.7)
Knee Extension Torque (6-month)	112.3 (31.7)	NA
Knee Extension Torque (12-month)	122.2 (30.1)	128.0 (40.4)
Knee Flexion Torque (baseline)	64.5 (27.0)	72.6 (11.3)
Knee Flexion Torque (6-month)	82.3 (27.7)	NA
Knee Flexion Torque (12-month)	86.1 (23.5)	94.0 (17.3)
KOOS Sport (baseline)	37.5 (22.3)	25.5 (30.1)
KOOS Sport (12-month)	63.4 (26.8)	46.9 (35.9)
KOOS QoL (baseline)	54.9 (27.0)	34.2 (39.8)
KOOS QoL (6-month)	77.9 (21.6)	NA
KOOS QoL (12-month)	84.3 (19.5)	66.3 (39.0)
KOOS Symptoms (baseline)	65.0 (18.0)	59.1 (22.2)
KOOS Symptoms (6-month)	82.2 (12.9)	NA
KOOS Symptoms (12-month)	82.2 (14.8)	81.3 (26.0)
YBT Composite Reach (baseline)	231.8 (22.4)	247.0 (29.9)
YBT Composite Reach (6-month)	240.8 (27.2)	NA
YBT Composite Reach (12-month)	253.1 (23.1)	272.7 (21.7)
Triple Hop (baseline	338.1 (69.8)	292.5 (50.5)
Triple Hop (6-months)	336.0 (72.5)	NA
Triple Hop (12-months)	348.9 (71.2)	NA
Age (baseline)	16.3 (1.8)	16.5 (2.0)
Height (baseline)	169.0 (8.9)	174.1 (10.0)
Weight (baseline)	67.8 (17.4)	75.3 (18.8)
Sex		
F	60	3
M	32	11
Injury Type		
ACL	52	8
Non-ACL	40	6
Family Income	128,258.2 (30,586.9)	132,943.4 (238,23.0)
Weekly Sport Exposure	447.3 (362.6)	546.7 (186.1)
Limb Dominance of Injured Leg		
Dominant	55	10
Non-Dominant	37	4
Treatment (baseline)		
Surgery and PT	6	4
Competition Level (baseline)		
Recreational	6	0
Competitive	66	12
Elite	18	1
Sport Type (baseline)		
Collision	29	9
Field/Court	53	5
Individual	10	0

ACL (anterior cruciate ligament), F (female), KOOS (Knee injury and Osteoarthritis Outcome Score), M (male), PT (physiotherapy), QoL (quality of life), YBT (Y-Balance Test)

Table 5. Missing Data Assessment for the KOOS Sport at 12-months

Variable	Not Missing (n=86)	Missing (N=20)
Knee Extension Torque (baseline)	90.1 (36.3)	92.2 (39.8)
Knee Extension Torque (6-month)	113.3 (32.7)	110.8 (28.7)
Knee Extension Torque (12-month)	122.3 (30.4)	NA
Knee Flexion Torque (baseline)	64.7 (26.4)	67.2 (26.4)
Knee Flexion Torque (6-month)	82.7 (28.2)	83.1 (25.4)
Knee Flexion Torque (12-month)	86.6 (23.4)	NA
KOOS Sport (baseline)	34.9 (22.3)	41.1 (28.8)
KOOS Sport (6-month)	55.7 (26.8)	62.5 (27.0)
KOOS Sport (12-month)	NA	NA
KOOS QoL (baseline)	51.9 (28.2)	54.2 (35.7)
KOOS QoL (6-month)	77.5 (21.9)	81.5 (19.7)
KOOS QoL (12-month)	83.4 (20.8)	NA
KOOS Symptoms (baseline)	63.9 (16.9)	65.8 (25.0)
KOOS Symptoms (6-month)	81.8 (12.1)	85.7 (18.4)
KOOS Symptoms (12-month)	82.2 (15.3)	NA
YBT Composite Reach (baseline)	232.7 (23.1)	235.6 (25.5)
YBT Composite Reach (6-month)	242.4 (27.4)	233.3 (27.4)
YBT Composite Reach (12-month)	253.7 (23.4)	NA
Triple Hop (baseline	332.1 (70.2)	335.9 (69.3)
Triple Hop (6-months)	342.2 (67.7)	316.8 (122.8)
Triple Hop (12-months)	348.6 (70.5)	NA
Age (baseline)	16.3 (1.8)	16.5 (2.1)
Height (baseline)	169.8 (9.4)	169.3 (8.0)
Weight (baseline)	68.0 (16.9)	72.2 (20.8)
Sex		
F	54	9
M	32	11
Injury Type		
ACL	51	9
Non-ACL	35	11
Family Income	127,970.8 (30,629.0)	132,773.5 (25,818.2)
Weekly Sport Exposure	446.5 (374.2)	513.2 (193.4)
Limb Dominance of Injured Leg		
Dominant	51	14
Non-Dominant	35	6
Treatment (baseline)		
Surgery and PT	9	1
Competition Level (baseline)		
Recreational	5	1
Competitive	69	18
Elite	19	0
Sport Type (baseline)		
Collision	27	11
Field/Court	49	9
Individual	10	0

ACL (anterior cruciate ligament), F (female), KOOS (Knee injury and Osteoarthritis Outcome Score), M (male), PT (physiotherapy), QoL (quality of life), YBT (Y-Balance Test)

Table 6. Missing Data Assessment for the YBT at Baseline

Variable	Not Missing (n=75)	Missing (N=31)
Knee Extension Torque (baseline)	89.6 (37.8)	95.3 (30.4)
Knee Extension Torque (6-month)	109.2 (32.6)	124.8 (28.1)
Knee Extension Torque (12-month)	120.8 (29.3)	126.4 (32.8)
Knee Flexion Torque (baseline)	64.6 (26.5)	67.7 (25.7)
Knee Flexion Torque (6-month)	82.0 (28.3)	84.8 (26.7)
Knee Flexion Torque (12-month)	85.6 (23.0)	88.7 (24.4)
KOOS Sport (baseline)	40.4 (24.5)	25.6 (17.8)
KOOS Sport (6-month)	59.0 (25.8)	49.2 (28.4)
KOOS Sport (12-month)	65.8 (26.5)	54.5 (28.1)
KOOS QoL (baseline)	59.3 (25.4)	35.8 (32.2)
KOOS QoL (6-month)	80.4 (19.3)	71.0 (26.4)
KOOS QoL (12-month)	83.5 (20.6)	83.3 (21.6)
KOOS Symptoms (baseline)	68.7 (16.8)	53.7 (18.4)
KOOS Symptoms (6-month)	82.1 (12.8)	82.6 (13.3)
KOOS Symptoms (12-month)	82.6 (14.1)	81.1 (18.1)
YBT Composite Reach (baseline)	NA	NA
YBT Composite Reach (6-month)	242.8 (24.7)	236.9 (34.5)
YBT Composite Reach (12-month)	252.7 (22.5)	256.8 (25.3)
Triple Hop (baseline	332.9 (68.7)	NA
Triple Hop (6-months)	344.0 (70.8)	317.2 (93.7)
Triple Hop (12-months)	351.5 (72.9)	334.8 (59.4)
Age (baseline)	16.2 (1.8)	16.6 (1.8)
Height (baseline)	168.1 (8.1)	173.5 (10.4)
Weight (baseline)	66.4 (15.0)	74.6 (22.2)
Sex		
F	49	14
M	26	17
Injury Type		
ACL	37	23
Non-ACL	38	8
Family Income	128,499.8 (30,840.0)	129,789.6 (27,302.4)
Weekly Sport Exposure	445.3 (261.0)	493.1 (499.4)
Limb Dominance of Injured Leg		
Dominant	46	19
Non-Dominant	29	12
Treatment (baseline)		
Surgery and PT	4	6
Competition Level (baseline)		
Recreational	5	1
Competitive	53	25
Elite	14	5
Sport Type (baseline)		
Collision	24	14
Field/Court	42	16
Individual	9	1

ACL (anterior cruciate ligament), F (female), KOOS (Knee injury and Osteoarthritis Outcome Score), M (male), PT (physiotherapy), QoL (quality of life), YBT (Y-Balance Test)

Table 7. Missing Data Assessment for the YBT at 6-months

Variable	Not Missing (n=84)	Missing (N=22)
Knee Extension Torque (baseline)	89.6 (35.9)	94.7 (41.5)
Knee Extension Torque (6-month)	113.3 (32.2)	NA
Knee Extension Torque (12-month)	121.0 (29.0)	133.1 (38.6)
Knee Flexion Torque (baseline)	65.1 (26.9)	65.3 (23.2)
Knee Flexion Torque (6-month)	81.7 (26.6)	NA
Knee Flexion Torque (12-month)	86.1 (23.6)	88.5 (21.4)
KOOS Sport (baseline)	37.3 (22.3)	31.0 (28.1)
KOOS Sport (6-month)	56.5 (26.9)	56.3 (26.7)
KOOS Sport (12-month)	63.0 (27.3)	60.4 (28.2)
KOOS QoL (baseline)	55.9 (26.9)	38.1 (35.5)
KOOS QoL (6-month)	78.4 (21.6)	73.9 (22.9)
KOOS QoL (12-month)	84.3 (20.1)	78.3 (25.0)
KOOS Symptoms (baseline)	65.2 (18.0)	60.4 (20.6)
KOOS Symptoms (6-month)	82.5 (12.7)	79.7 (14.8)
KOOS Symptoms (12-month)	82.1 (15.0)	82.5 (17.3)
YBT Composite Reach (baseline)	232.6 (22.3)	236.4 (29.5)
YBT Composite Reach (6-month)	NA	NA
YBT Composite Reach (12-month)	235.9 (22.7)	253.9 (28.6)
Triple Hop (baseline	334.3 (64.6)	326.6 (92.6)
Triple Hop (6-months)	339.2 (75.0)	NA
Triple Hop (12-months)	349.6 (70.3)	341.3 (78.5)
Age (baseline)	16.3 (1.8)	16.4 (2.0)
Height (baseline)	169.2 (8.9)	171.8 (9.9)
Weight (baseline)	67.5 (15.9)	74.0 (22.9)
Sex		
F	55	8
M	29	14
Injury Type		
ACL	49	11
Non-ACL	35	11
Family Income	127,806.5 (31,490.4)	132,964.4 (21,832.9)
Weekly Sport Exposure	442.8 (370.2)	524.5 (287.0)
Limb Dominance of Injured Leg		
Dominant	52	13
Non-Dominant	32	9
Treatment (baseline)		
Surgery and PT	4	6
Competition Level (baseline)		
Recreational	6	0
Competitive	59	19
Elite	17	2
Sport Type (baseline)		
Collision	27	11
Field/Court	48	10
Individual	9	1

ACL (anterior cruciate ligament), F (female), KOOS (Knee injury and Osteoarthritis Outcome Score), M (male), PT (physiotherapy), QoL (quality of life), YBT (Y-Balance Test)

Table 8. Missing Data Assessment for the YBT at 12-months

Variable	Not Missing (n=77)	Missing (N=29)
Knee Extension Torque (baseline)	91.7 (37.0)	86.8 (36.3)
Knee Extension Torque (6-month)	112.3 (33.5)	116.0 (25.5)
Knee Extension Torque (12-month)	122.4 (30.2)	NA
Knee Flexion Torque (baseline)	66.3 (26.1)	61.8 (27.0)
Knee Flexion Torque (6-month)	83.1 (26.1)	61.8 (27.0)
Knee Flexion Torque (12-month)	86.4 (23.2)	NA
KOOS Sport (baseline)	36.7 (22.3)	34.2 (27.1)
KOOS Sport (6-month)	55.4 (26.6)	60.8 (27.4)
KOOS Sport (12-month)	63.5 (25.7)	55.7 (38.2)
KOOS QoL (baseline)	53.6 (28.4)	48.8 (32.7)
KOOS QoL (6-month)	77.4 (22.5)	80.3 (17.9)
KOOS QoL (12-month)	84.8 (18.8)	73.0 (31.4)
KOOS Symptoms (baseline)	64.3 (17.3)	63.9 (21.8)
KOOS Symptoms (6-month)	81.8 (12.7)	84.1 (13.8)
KOOS Symptoms (12-month)	82.1 (14.9)	82.9 (18.4)
YBT Composite Reach (baseline)	233.0 (22.1)	233.8 (27.3)
YBT Composite Reach (6-month)	241.8 (28.0)	239.2 (25.5)
YBT Composite Reach (12-month)	NA	NA
Triple Hop (baseline	329.0 (70.6)	345.6 (66.4)
Triple Hop (6-months)	344.6 (68.8)	319.3 (95.0)
Triple Hop (12-months)	348.6 (70.5)	NA
Age (baseline)	16.3 (1.8)	16.4 (1.9)
Height (baseline)	169.8 (9.6)	169.4 (7.9)
Weight (baseline)	68.1 (17.5)	70.9 (18.3)
Sex		
F	48	15
M	29	14
Injury Type		
ACL	47	13
Non-ACL	30	16
Family Income	128,171.7 (31,209.3)	130,749.6 (25,784.3)
Weekly Sport Exposure	416.6 (253.9)	572.2 (506.7)
Limb Dominance of Injured Leg		
Dominant	48	17
Non-Dominant	29	12
Treatment (baseline)		
Surgery and PT	6	4
Competition Level (baseline)		
Recreational	5	1
Competitive	53	25
Elite	17	2
Sport Type (baseline)		
Collision	24	14
Field/Court	44	14
Individual	9	1

ACL (anterior cruciate ligament), F (female), KOOS (Knee injury and Osteoarthritis Outcome Score), M (male), PT (physiotherapy), QoL (quality of life), YBT (Y-Balance Test)

Table 9. Missing Data Assessment for the Knee Extension Strength at Baseline

Variable	Not Missing (n=87)	Missing (N=19)
Knee Extension Torque (baseline)	NA	NA
Knee Extension Torque (6-month)	111.5 (32.1)	122.4 (32.2)
Knee Extension Torque (12-month)	124.3 (30.4)	112.9 (28.4)
Knee Flexion Torque (baseline)	65.1 (26.2)	NA
Knee Flexion Torque (6-month)	82.0 (28.3)	86.7 (24.9)
Knee Flexion Torque (12-month)	87.1 (23.4)	83.3 (23.2)
KOOS Sport (baseline)	38.6 (24.0)	24.4 (17.8)
KOOS Sport (6-month)	57.4 (25.5)	50.0 (34.2)
KOOS Sport (12-month)	63.8 (26.3)	55.8 (32.4)
KOOS QoL (baseline)	56.1 (27.2)	35.5 (34.0)
KOOS QoL (6-month)	77.9 (21.0)	77.9 (26.2)
KOOS QoL (12-month)	84.2 (19.6)	78.8 (26.9)
KOOS Symptoms (baseline)	66.8 (17.7)	52.8 (18.4)
KOOS Symptoms (6-month)	81.8 (12.5)	84.8 (15.3)
KOOS Symptoms (12-month)	82.5 (13.9)	80.2 (21.9)
YBT Composite Reach (baseline)	232.6 (23.1)	NA
YBT Composite Reach (6-month)	239.5 (27.4)	253.0 (25.7)
YBT Composite Reach (12-month)	253.5 (22.1)	255.9 (29.1)
Triple Hop (baseline	332.8 (68.7)	NA
Triple Hop (6-months)	337.9 (72.6)	350.8 (103.7)
Triple Hop (12-months)	350.7 (70.3)	329.3 (78.2)
Age (baseline)	16.3 (1.8)	16.5 (1.8)
Height (baseline)	169.0 (8.7)	172.7 (10.7)
Weight (baseline)	67.7 (16.2)	73.8 (23.3)
Sex		
F	56	7
M	31	12
Injury Type		
ACL	47	13
Non-ACL	40	6
Family Income	128,654.6 (31,505.8)	129,895.2 (20,265.0)
Weekly Sport Exposure	458.6 (369.0)	463.5 (219.3)
Limb Dominance of Injured Leg		
Dominant	52	13
Non-Dominant	35	6
Treatment (baseline)		
Surgery and PT	4	6
Competition Level (baseline)		
Recreational	6	0
Competitive	62	16
Elite	16	3
Sport Type (baseline)		
Collision	28	10
Field/Court	50	8
Individual	9	1

ACL (anterior cruciate ligament), F (female), KOOS (Knee injury and Osteoarthritis Outcome Score), M (male), PT (physiotherapy), QoL (quality of life), YBT (Y-Balance Test)

Table 10. Missing Data Assessment for the Knee Extension Strength at 6-months

Variable	Not Missing (n=82)	Missing (N=24)
Knee Extension Torque (baseline)	89.4 (37.0)	95.0 (35.7)
Knee Extension Torque (6-month)	NA	NA
Knee Extension Torque (12-month)	121.4 (30.0)	129.2 (32.5)
Knee Flexion Torque (baseline)	64.8 (27.3)	66.5 (21.2)
Knee Flexion Torque (6-month)	82.7 (27.7)	NA
Knee Flexion Torque (12-month)	86.3 (23.7)	87.1 (21.4)
KOOS Sport (baseline)	37.2 (22.7)	31.8 (26.7)
KOOS Sport (6-month)	57.1 (26.9)	51.7 (26.2)
KOOS Sport (12-month)	63.0 (27.6)	60.6 (25.9)
KOOS QoL (baseline)	54.9 (26.5)	43.0 (37.5)
KOOS QoL (6-month)	78.7 (20.6)	72.3 (28.6)
KOOS QoL (12-month)	84.0 (20.3)	80.0 (23.6)
KOOS Symptoms (baseline)	64.3 (17.8)	64.0 (21.5)
KOOS Symptoms (6-month)	82.2 (12.8)	82.5 (14.0)
KOOS Symptoms (12-month)	81.8 (15.3)	84.3 (15.7)
YBT Composite Reach (baseline)	232.4 (22.5)	236.9 (28.1)
YBT Composite Reach (6-month)	241.2 (27.8)	244.9 (13.3)
YBT Composite Reach (12-month)	252.4 (22.6)	256.7 (28.1)
Triple Hop (baseline	334.3 (64.6)	326.6 (92.6)
Triple Hop (6-months)	339.8 (75.6)	NA
Triple Hop (12-months)	352.0 (67.8)	323 (92.0)
Age (baseline)	16.2 (1.7)	16.8 (2.1)
Height (baseline)	169.0 (8.8)	172.2 (9.9)
Weight (baseline)	67.6 (16.2)	73.1 (21.7)
Sex		
F	55	8
M	27	16
Injury Type		
ACL	47	8
Non-ACL	35	16
Family Income	128,723.8 (31,318.6)	129,400.5 (24,067.9)
Weekly Sport Exposure	456.7 (380.7)	469.5 (179.2)
Limb Dominance of Injured Leg		
Dominant	50	15
Non-Dominant	32	9
Treatment (baseline)		
Surgery and PT	4	6
Competition Level (baseline)		
Recreational	5	1
Competitive	59	19
Elite	17	2
Sport Type (baseline)		
Collision	27	11
Field/Court	45	13
Individual	10	0

ACL (anterior cruciate ligament), F (female), KOOS (Knee injury and Osteoarthritis Outcome Score), M (male), PT (physiotherapy), QoL (quality of life), YBT (Y-Balance Test)

Table 11. Missing Data Assessment for the Knee Extension Strength at 12-months

Variable	Not Missing (n=77)	Missing (N=29)
Knee Extension Torque (baseline)	91.7 (37.0)	86.8 (36.3)
Knee Extension Torque (6-month)	112.3 (33.5)	116.0 (25.5)
Knee Extension Torque (12-month)	NA	NA
Knee Flexion Torque (baseline)	66.3 (26.1)	61.8 (27.0)
Knee Flexion Torque (6-month)	83.1 (28.5)	81.2 (25.0)
Knee Flexion Torque (12-month)	86.4 (23.2)	NA
KOOS Sport (baseline)	36.7 (22.3)	34.2 (27.1)
KOOS Sport (6-month)	55.4 (26.6)	60.8 (27.4)
KOOS Sport (12-month)	63.5 (25.7)	55.7 (38.2)
KOOS QoL (baseline)	53.6 (28.4)	48.8 (32.7)
KOOS QoL (6-month)	77.4 (22.5)	80.3 (17.9)
KOOS QoL (12-month)	84.8 (18.8)	73.0 (31.4)
KOOS Symptoms (baseline)	64.3 (17.3)	63.9 (21.8)
KOOS Symptoms (6-month)	81.8 (12.7)	84.1 (13.8)
KOOS Symptoms (12-month)	82.1 (14.9)	82.9 (18.4)
YBT Composite Reach (baseline)	233.0 (22.1)	233.8 (27.3)
YBT Composite Reach (6-month)	241.8 (28.0)	239.2 (25.5)
YBT Composite Reach (12-month)	253.9 (23.2)	NA
Triple Hop (baseline	329.0 (70.6)	345.6 (66.4)
Triple Hop (6-months)	344.8 (68.8)	319.3 (95.0)
Triple Hop (12-months)	348.6 (70.5)	NA
Age (baseline)	16.3 (1.8)	16.4 (1.9)
Height (baseline)	169.8 (9.6)	169.4 (7.9)
Weight (baseline)	68.1 (17.5)	70.9 (18.3)
Sex		
F	48	15
M	29	14
Injury Type		
ACL	47	13
Non-ACL	30	16
Family Income	128,171.7 (31,209.3)	130,749.6 (25,784.3)
Weekly Sport Exposure	416.6 (253.9)	572.2 (506.7)
Limb Dominance of Injured Leg		
Dominant	48	17
Non-Dominant	29	12
Treatment (baseline)		
Surgery and PT	6	4
Competition Level (baseline)		
Recreational	5	1
Competitive	53	25
Elite	17	2
Sport Type (baseline)		
Collision	24	14
Field/Court	44	14
Individual	9	1

ACL (anterior cruciate ligament), F (female), KOOS (Knee injury and Osteoarthritis Outcome Score), M (male), PT (physiotherapy), QoL (quality of life), YBT (Y-Balance Test)

Table 12. Missing Data Assessment for the Knee Flexion Strength at Baseline

Variable	Not Missing (n=85)	Missing (N=21)
Knee Extension Torque (baseline)	88.8 (35.3)	158.0 (39.1)
Knee Extension Torque (6-month)	111.3 (32.2)	123.2 (30.9)
Knee Extension Torque (12-month)	123.9 (30.5)	115.6 (29.1)
Knee Flexion Torque (baseline)	NA	NA
Knee Flexion Torque (6-month)	82.0 (28.3)	86.7 (24.9)
Knee Flexion Torque (12-month)	86.8 (23.5)	84.9 (22.9)
KOOS Sport (baseline)	37.8 (23.7)	28.9 (22.3)
KOOS Sport (6-month)	57.0 (25.4)	53.4 (35.0)
KOOS Sport (12-month)	63.5 (26.3)	58.1 (32.3)
KOOS QoL (baseline)	55.2 (26.9)	41.0 (36.6)
KOOS QoL (6-month)	77.7 (21.0)	79.6 (25.8)
KOOS QoL (12-month)	84.2 (19.7)	79.3 (25.9)
KOOS Symptoms (baseline)	66.1 (17.4)	56.8 (21.5)
KOOS Symptoms (6-month)	81.7 (12.5)	85.4 (14.9)
KOOS Symptoms (12-month)	82.4 (14.0)	81.1 (21.3)
YBT Composite Reach (baseline)	232.0 (23.1)	262.0 (9.3)
YBT Composite Reach (6-month)	239.2 (27.4)	253.7 (24.6)
YBT Composite Reach (12-month)	253.4 (22.3)	256.1 (28.0)
Triple Hop (baseline	330.1 (68.2)	366.3 (93.0)
Triple Hop (6-months)	336.1 (72.5)	361.7 (96.5)
Triple Hop (12-months)	350.7 (70.3)	329.3 (78.2)
Age (baseline)	16.3 (1.9)	16.5 (1.7)
Height (baseline)	169.1 (8.8)	172.4 (10.3)
Weight (baseline)	67.9 (16.3)	72.7 (22.4)
Sex		
F	55	8
M	30	13
Injury Type		
ACL	47	13
Non-ACL	38	8
Family Income	128,260.4 (31,625.2)	131,372.6 (20,742.1)
Weekly Sport Exposure	456.5 (372.4)	471.6 (216.6)
Limb Dominance of Injured Leg		
Dominant	50	15
Non-Dominant	35	6
Treatment (baseline)		
Surgery and PT	4	6
Competition Level (baseline)		
Recreational	6	0
Competitive	60	18
Elite	16	3
Sport Type (baseline)		
Collision	27	11
Field/Court	49	9
Individual	9	1

ACL (anterior cruciate ligament), F (female), KOOS (Knee injury and Osteoarthritis Outcome Score), M (male), PT (physiotherapy), QoL (quality of life), YBT (Y-Balance Test)

Table 13. Missing Data Assessment for the Knee Flexion Strength at 6-months

Variable	Not Missing (n=77)	Missing (N=29)
Knee Extension Torque (baseline)	89.1 (37.9)	94.4 (32.9)
Knee Extension Torque (6-month)	112.4 (32.2)	123.1 (32.8)
Knee Extension Torque (12-month)	120.7 (29.7)	129.5 (32.2)
Knee Flexion Torque (baseline)	64.3 (27.6)	67.7 (21.3)
Knee Flexion Torque (6-month)	NA	NA
Knee Flexion Torque (12-month)	86.5 (24.0)	85.9 (20.4)
KOOS Sport (baseline)	36.1 (21.6)	35.7 (28.7)
KOOS Sport (6-month)	56.5 (26.9)	56.3 (26.9)
KOOS Sport (12-month)	62.3 (27.8)	63.6 (26.0)
KOOS QoL (baseline)	54.5 (26.4)	46.3 (36.9)
KOOS QoL (6-month)	78.2 (21.0)	76.9 (25.1)
KOOS QoL (12-month)	84.4 (20.5)	79.7 (22.0)
KOOS Symptoms (baseline)	63.9 (17.5)	65.1 (21.4)
KOOS Symptoms (6-month)	82.2 (12.8)	82.4 (13.3)
KOOS Symptoms (12-month)	81.8 (15.3)	83.5 (15.5)
YBT Composite Reach (baseline)	232.2 (22.6)	236.2 (26.4)
YBT Composite Reach (6-month)	240.6 (28.2)	248.5 (18.1)
YBT Composite Reach (12-month)	253.0 (23.0)	257.4 (24.5)
Triple Hop (baseline	328.5 (63.7)	344.7 (85.2)
Triple Hop (6-months)	337.1 (75.5)	54.4 (76.2)
Triple Hop (12-months)	347.2 (65.6)	355.6 (95.0)
Age (baseline)	16.2 (1.7)	16.6 (2.1)
Height (baseline)	169.1 (8.9)	171.4 (9.7)
Weight (baseline)	68.0 (16.5)	71.0 (20.7)
Sex		
F	52	11
M	25	18
Injury Type		
ACL	45	15
Non-ACL	32	14
Family Income	128,332.3 (31,954.9)	130,323.2 (23,216.6)
Weekly Sport Exposure	457.2 (393.2)	465.8 (165.4)
Limb Dominance of Injured Leg		
Dominant	45	20
Non-Dominant	32	9
Treatment (baseline)		
Surgery and PT	5	5
Competition Level (baseline)		
Recreational	5	1
Competitive	56	22
Elite	15	4
Sport Type (baseline)		
Collision	25	13
Field/Court	42	16
Individual	10	0

ACL (anterior cruciate ligament), F (female), KOOS (Knee injury and Osteoarthritis Outcome Score), M (male), PT (physiotherapy), QoL (quality of life), YBT (Y-Balance Test)

Table 14. Missing Data Assessment for the Knee Extension Strength at 12-months

Variable	Not Missing (n=72)	Missing (N=34)
Knee Extension Torque (baseline)	91.5 (38.2)	88.1 (33.6)
Knee Extension Torque (6-month)	109.5 (32.6)	124.0 (28.6)
Knee Extension Torque (12-month)	120.3 (29.7)	152.7 (20.6)
Knee Flexion Torque (baseline)	65.3 (26.4)	64.8 (26.3)
Knee Flexion Torque (6-month)	81.7 (27.3)	86.0 (29.6)
Knee Flexion Torque (12-month)	NA	NA
KOOS Sport (baseline)	37.5 (22.6)	32.8 (25.7)
KOOS Sport (6-month)	54.9 (26.9)	61.2 (26.3)
KOOS Sport (12-month)	63.1 (26.0)	60.5 (33.5)
KOOS QoL (baseline)	53.4 (28.3)	50.0 (32.2)
KOOS QoL (6-month)	76.2 (22.7)	83.3 (17.4)
KOOS QoL (12-month)	84.3 (19.1)	79.3 (27.7)
KOOS Symptoms (baseline)	64.3 (17.6)	64.1 (20.8)
KOOS Symptoms (6-month)	81.5 (12.7)	84.5 (13.4)
KOOS Symptoms (12-month)	81.6 (15.1)	84.8 (16.5)
YBT Composite Reach (baseline)	233.4 (22.7)	232.7 (25.5)
YBT Composite Reach (6-month)	241.0 (28.5)	242.2 (24.4)
YBT Composite Reach (12-month)	253.6 (23.5)	257.7 (20.1)
Triple Hop (baseline	329.6 (72.5)	341.8 (61.4)
Triple Hop (6-months)	340.2 (68.5)	336.8 (91.0)
Triple Hop (12-months)	344.3 (71.0)	389.2 (56.7)
Age (baseline)	16.3 (1.8)	16.5 (1.8)
Height (baseline)	169.5 (9.5)	170.2 (8.5)
Weight (baseline)	67.7 (17.7)	71.2 (17.7)
Sex		
F	46	17
M	26	17
Injury Type		
ACL	44	16
Non-ACL	28	18
Family Income	128,866.8 (31,55.8)	128,898.7 (25,867.5)
Weekly Sport Exposure	424.9 (256.4)	530.6 (480.2)
Limb Dominance of Injured Leg		
Dominant	44	21
Non-Dominant	28	13
Treatment (baseline)		
Surgery and PT	5	5
Competition Level (baseline)		
Recreational	5	1
Competitive	49	29
Elite	16	3
Sport Type (baseline)		
Collision	21	17
Field/Court	42	16
Individual	9	1

ACL (anterior cruciate ligament), F (female), KOOS (Knee injury and Osteoarthritis Outcome Score), M (male), PT (physiotherapy), QoL (quality of life), YBT (Y-Balance Test)

Appendix 5: Full models and sensitivity analysis results

Table 1. Outcome Triple Hop Distance (cm) 6 to 12-month follow-up

Parameter	Coefficient	Model based 95%CI
Intercept	116.9	-374.9, 608.7
Extensor Strength (12-month)	0.9	-0.5, 2.3
Extensor Strength (6-month)	-0.5	-1.5, 0.5
Sex (male)	40.9	-7.8, 89.5
Age (baseline)	0.6	-9.7, 10.9
Weight (baseline)	-1.0	-2.8, 0.9
Height (baseline)	0.1	-3.5, 3.7
Injured Limb (dominant)	-11.9	-52.9, 29.0
Treatment (baseline)		
Surgery + PT	38.1	-45.3, 121.6
PT only	26.4	-35.9, 88.7
Triple Hop distance (6-month)	0.5	0.1, 0.9

PT (physiotherapy), CI (confidence interval)

Table 2. Outcome Triple Hop Distance (cm) 6 to 12-month follow-up

Parameter	Coefficient	Model based 95%CI
Intercept	90.4	-391.6, 572.5
Flexor Strength (12-month)	0.3	-1.0, 1.7
Flexor Strength (6-month)	-0.2	-1.4, 1.1
Sex (male)	42.6	-11.3, 96.4
Age (baseline)	0.2	-11.1, 11.5
Weight (baseline)	-0.9	-2.9, 1.2
Height (baseline)	0.5	-3.1, 4.2
Injured Limb (dominant)	-6.3	-49.1, 36.4
Treatment (baseline)		
Surgery + PT	28.0	-65.5, 121.6
PT only	23.7	-45.5, 93.0
Triple Hop distance (6-month)	0.5	0.1, 0.9

PT (physiotherapy), CI (confidence interval)

Table 3. Outcome Y-Balance Test Distance (cm) baseline to 6-month follow-up

Parameter	Coefficient	Model based 95%CI
Intercept	91.5	-80.4, 263.4
Extensor Strength (6-month)	0.2	-0.2, 0.6
Extensor Strength (baseline)	0.1	-0.2, 0.3
Sex (male)	-0.1	-19.9, 19.8
Age (baseline)	0.6	-3.5, 4.6
Weight (baseline)	-0.2	-0.8, 0.4
Height (baseline)	0.5	-0.7, 1.7
Injured Limb (dominant)	-3.3	-16.4, 9.7
Treatment (baseline)		
Surgery + PT	0.3	-31.3, 32.0
PT only	-5.7	-28.2, 16.7
YBT reach distance (Baseline)	0.2	-0.2, 0.6

PT (physiotherapy), YBT (Y-Balance Test), CI (confidence interval)

Table 4. Outcome Y-Balance Test Distance (cm) 6 to 12-month follow-up

Parameter	Coefficient	Model based 95%CI
Intercept	-1.3	-203.8, 201.3
Extensor Strength (12-month)	0.1	-0.4, 0.5
Extensor Strength (6-month)	-0.1	-0.5, 0.3
Sex (male)	6.6	-13.6, 26.8
Age (baseline)	0.3	-4.4, 4.9
Weight (baseline)	-0.3	-0.9, 0.3
Height (baseline)	1.1	-0.2, 2.5
Injured Limb (dominant)	-1.5	-17.4, 14.4
Treatment (baseline)		
Surgery + PT	1.0	-30.1, 32.1
PT only	8.1	-16.4, 32.6
YBT reach distance (6-month)	0.3	-0.1, 0.6

PT (physiotherapy), YBT (Y-Balance Test), CI (confidence interval)

Table 5. Outcome Y-Balance Test Distance (cm) baseline to 12-month follow-up

Parameter	Coefficient	Model based 95%CI
Intercept	21.2	-170.6, 212.9
Extensor Strength (12-month)	0.1	-0.4, 0.5
Extensor Strength (baseline)	0.0	-0.3, 0.3
Sex (male)	4.9	-16.4, 26.2
Age (baseline)	0.2	-5.0, 5.3
Weight (baseline)	-0.4	-1.0, 0.3
Height (baseline)	1.2	-0.2, 0.6
Injured Limb (dominant)	-2.1	-18.1, 13.9
Treatment (baseline)		
Surgery + PT	1.4	-30.4, 33.3
PT only	4.9	-20.3, 30.0
YBT reach distance (Baseline)	0.2	-0.3, 0.6

PT (physiotherapy), YBT (Y-Balance Test), CI (confidence interval)

Table 6. Outcome Y-Balance Test Distance (cm) baseline to 6-month follow-up

Parameter	Coefficient	Model based 95%CI
Intercept	92.0	-78.4, 262.4
Flexor Strength (6-month)	0.3	-0.1, 0.8
Flexor Strength (baseline)	0.1	-0.2, 0.4
Sex (male)	-3.0	-22.4, 16.4
Age (baseline)	0.8	-3.0, 4.7
Weight (baseline)	-0.3	-0.9, 0.3
Height (baseline)	0.4	-0.7, 1.6
Injured Limb (dominant)	-4.3	-17.0, 8.4
Treatment (baseline)		
Surgery + PT	4.0	-28.4, 36.4
PT only	-3.9	-25.9, 18.1
YBT reach distance (Baseline)	0.2	-0.1, 0.6

PT (physiotherapy), YBT (Y-Balance Test), CI (confidence interval)

Table 7. Outcome Y-Balance Test Distance (cm) 6 to 12-month follow-up

Parameter	Coefficient	Model based 95%CI
Intercept	9.4	-177.1, 195.8
Flexor Strength (12-month)	0.0	-0.7, 0.7
Flexor Strength (6-month)	0.0	-0.5, 0.5
Sex (male)	6.5	-14.5, 27.6
Age (baseline)	0.2	-4.3, 4.7
Weight (baseline)	-0.3	-0.9, 0.3
Height (baseline)	1.1	-0.3, 2.4
Injured Limb (dominant)	-1.1	-16.5, 14.4
Treatment (baseline)		
Surgery + PT	0.5	-29.5, 30.5
PT only	7.5	-17.2, 32.2
YBT reach distance (6-month)	0.3	-0.1, 0.7

PT (physiotherapy), YBT (Y-Balance Test), CI (confidence interval)

Table 8. Outcome Y-Balance Test Distance (cm) at 12-month follow-up

Parameter	Coefficient	Model based 95%CI
Intercept	17.5	-180.3, 215.3
Flexor Strength (12-month)	0.0	-0.6, 0.7
Flexor Strength (baseline)	0.0	-0.4, 0.4
Sex (male)	5.0	-16.6, 26.6
Age (baseline)	0.2	-4.7, 5.0
Weight (baseline)	-0.3	-1.0, 0.3
Height (baseline)	1.2	-0.1, 2.5
Injured Limb (dominant)	-1.6	-17.5, 14.4
Treatment (baseline)		
Surgery + PT	0.1	-31.6, 31.8
PT only	4.4	-20.2, 29.1
YBT reach distance (Baseline)	0.2	-0.2, 0.6

PT (physiotherapy), YBT (Y-Balance Test), CI (confidence interval)

Table 9. Outcome KOOS$_{sport}$ (0-1) baseline to 6-month follow-up

Parameter	Coefficient*	Model based 95%CI
Intercept	0.01	-2.5, 2.6
Extensor Strength (6-month)	0.001	0.00, 0.02
Extensor Strength (baseline)	-0.0001	-0.01, 0.01
Sex (male)	-0.1	-0.7, 0.5
Age (baseline)	-0.1	-0.3, 0.04
Sport exposure (baseline)	0.0004	0.00, 0.001
Income (baseline)	0.0	0.0, 0.0
Weight (baseline)	-0.01	-0.02, 0.01
Treatment (baseline)		
Surgery + PT	0.1	-0.9, 1.2
PT only	0.2	-0.6, 1.0
KOOS$_{sport}$ (Baseline)	2.4	1.2, 3.6

*Logit units, PT (physiotherapy), KOOS (Knee injury and Osteoarthritis Outcome Score), CI (confidence interval)

Table 10. Outcome KOOS$_{sport}$ (0-1) 6 to 12-month follow-up

Parameter	Coefficient*	Model based 95%CI
Intercept	-0.7	-3.4, 1.9
Extensor Strength (12-month)	0.02	0.0, 0.03
Extensor Strength (6-month)	0.00	-0.02, 0.00
Sex (male)	0.1	-0.5, 0.8
Age (baseline)	-0.05	-0.2, 0.1
Sport exposure (baseline)	0.00	0.00, 0.00
Income (baseline)	0.00	0.00, 0.00
Weight (baseline)	-0.0004	-0.02, 0.01
Treatment (baseline)		
Surgery + PT	-0.4	-0.8,1.5
PT only	0.1	-0.7,1.0
KOOS$_{sport}$ (6-month)	3.0	1.9, 4.1

*Logit units, PT (physiotherapy), KOOS (Knee injury and Osteoarthritis Outcome Score), CI (confidence interval)

Table 11. Outcome $KOOS_{sport}$ (0-1) baseline to 12-month follow-up

Parameter	Coefficient*	Model based 95%CI
Intercept	0.7	-2.4, 3.7
Extensor Strength (12-month)	0.01	0.00, 0.03
Extensor Strength (baseline)	-0.001	-0.01, 0.01
Sex (male)	-0.02	-0.7, 0.6
Age (baseline)	-0.1	-0.3, 0.1
Sport exposure (baseline)	-0.0004	0.00, 0.00
Income (baseline)	0.00	0.00, 0.00
Weight (baseline)	-0.01	-0.03, 0.01
Treatment (baseline)		
Surgery + PT	0.5	-0.7, 1.6
PT only	0.3	-0.6, 1.1
$KOOS_{sport}$ (Baseline)	1.5	0.3, 2.8

*Logit units, PT (physiotherapy), KOOS (Knee injury and Osteoarthritis Outcome Score), CI (confidence interval)

Table 12. Outcome $KOOS_{sport}$ (0-1) baseline to 6-month follow-up

Parameter	Coefficient*	Model based 95%CI
Intercept	0.2	-2.4, 2.8
Flexor Strength (6-month)	0.01	-0.01, 0.02
Flexor Strength (baseline)	0.01	-0.01, 0.02
Sex (male)	-0.1	-0.8, 0.5
Age (baseline)	-0.1	-0.2, 0.04
Sport exposure (baseline)	0.0004	0.00, 0.00
Income (baseline)	0.00	0.00, 0.00
Weight (baseline)	-0.01	-0.02, 0.01
Treatment (baseline)		
Surgery + PT	0.2	-0.9, 1.3
PT only	0.2	-0.6, 1.1
$KOOS_{sport}$ (Baseline)	2.3	1.2, 3.5

*Logit units, PT (physiotherapy), KOOS (Knee injury and Osteoarthritis Outcome Score), CI (confidence interval)

Table 13. Outcome $KOOS_{sport}$ (0-1) 6 to 12-month follow-up

Parameter	Coefficient*	Model based 95%CI
Intercept	-0.1	-2.9, 2.6
Flexor Strength (12-month)	0.00	0.00, 0.02
Flexor Strength (6-month)	0.00	-0.02, 0.01
Sex (male)	0.2	-0.5, 0.9
Age (baseline)	-0.1	-0.2, 0.01
Sport exposure (baseline)	0.00	0.00, 0.00
Income (baseline)	0.00	0.00, 0.00
Weight (baseline)	0.00	-0.02, 0.02
Treatment (baseline)		
Surgery + PT	0.2	-1.0, 1.3
PT only	0.1	-0.8,1.0
$KOOS_{sport}$ (6-month)	2.8	1.7, 4.0

*Logit units, PT (physiotherapy), KOOS (Knee injury and Osteoarthritis Outcome Score), CI (confidence interval)

Table 14. Outcome $KOOS_{sport}$ (0-1) baseline to 12-month follow-up

Parameter	Coefficient*	Model based 95%CI
Intercept	1.2	-1.8, 4.2
Flexor Strength (12-month)	0.01	-0.01, 0.02
Flexor Strength (baseline)	0.003	-0.01, 0.02
Sex (male)	0.03	-0.7, 0.7
Age (baseline)	-0.1	-0.3, 0.1
Sport exposure (baseline)	-0.0004	0.00, 0.00
Income (baseline)	0.00	0.00, 0.00
Weight (baseline)	-0.01	-0.02, 0.01
Treatment (baseline)		
Surgery + PT	0.3	-0.8, 1.5
PT only	0.2	-0.7, 1.1
$KOOS_{sport}$ (Baseline)	1.4	0.2, 2.7

*Logit units, PT (physiotherapy), KOOS (Knee injury and Osteoarthritis Outcome Score), CI (confidence interval)

Table 15. Exposure and Outcome Values in Observed Data

		Mean (SD)
Injured Limb, Knee Extensor Peak Torque		
Baseline		
	Raw (Nm)	90.4 (36.7)
	Normalized to BM (Nm/kg)	1.36 (0.52)
6-months		
	Raw (Nm)	113.0 (32.1)
	Normalized to BM (Nm/kg)	1.64 (0.41)
12-months		
	Raw (Nm)	122.4 (30.2)
	Normalized to BM (Nm/kg)	1.76 (0.37)
Injured Limb, Knee Flexor Peak Torque		
Baseline		
	Raw (Nm)	65.1 (26.2)
	Normalized to BM (Nm/kg)	0.97 (0.36)
6-months		
	Raw (Nm)	82.7 (27.7)
	Normalized to BM (Nm/kg)	1.19 (0.33)
12-months		
	Raw (Nm)	86.4 (23.2)
	Normalized to BM (Nm/kg)	1.25 (0.31)
Injured Limb, Triple Hop (cm)		
Baseline		332.8 (68.7)
6-months		339.1 (75.0)
12-months		348.6 (70.5)
$KOOS_{sport}$ (0-100)*		
Baseline		37.5 (18.8, 51.6)
6-months		56.3 (37.5, 75.0)
12-months		68.8 (43.8, 81.3)
Injured Limb, YBT Composite Reach (cm)		
Baseline		233.2 (23.4)
6-months		241.3 (27.4)
12-months		253.9 (23.2)

Values represent mean (standard deviation). BM (body mass), Nm (Newton-meter), kg (kilograms), YBT (Y-Balance Test)
*Data presented as median (interquartile limits)

Table 16. Sensitivity Analysis Results

Outcome	KOOS$_{sport}$ (0-100) AME (95%CI)*			Triple Hop Distance (cm) Beta (95%CI)	YBT Composite Reach Distance (cm) Beta (95%CI)		
Timeperiod**	0 to 6	6 to 12	0 to 12	6 to 12	0 to 6	6 to 12	0 to 12
**Knee Extension Torque as the Exposures**							
Normalized Strength (Nm/kg)[1]	10.3% (-3.1%, 23.6%)	23.2% (5.2%, 41.2%)	22.4% (5.3%, 39.6%)	77.0 (-16, 170)	14.1 (-9.6, 37.9)	9.9 (-22, 42)	8.9 (-18.9, 36.6)
Different Adjustment Set[2]	0.2% (0.0%, 0.4%)	0.3% (0.1%, 0.6%)	0.3% (0.0%, 0.6%)	1.0 (-0.5, 2.4)	0.2 (-0.2, 0.6)	0.1 (-0.4, 0.5)	0.1 (-0.4, 0.5)
Complete Case[3]	0.2% (0.1%, 0.4%)	0.3% (0.1%,0.5%)	0.4% (0.1%, 0.6%)	0.1 (-0.5, 0.7)	0.3 (0.1, 0.6)	0.1 (0.0, 0.3	-0.2 (-0.4, 0.1)
**Knee Flexion Torque as the Exposure**							
Normalized Strength (Nm/kg)[1]	9.0% (-6.6%, 24.7%)	8.5% (-10.1%, 27.1%)	13.3% (-3.3%, 29.9%)	36.3 (-54, 126)	24.9 (-2.2, 52.0)	5.8 (-38, 50)	7.4 (-29.3, 44.1)
Different Adjustment Set[2]	0.1% (-0.1%, 0.3%)	0.2% (-0.1%, 0.5%)	0.2% (-0.1%, 0.5%)	0.5 (-1.0, 1.9)	0.3 (-0.2, 0.7)	0.0 (-0.6, 0.7)	0.0 (-0.5, 0.6)
Complete Case[3]	0.1% (-0.1%, 0.3%)	0.3% (0.0%, 0.5%)	0.2% (-0.1%, 0.5%)	0.2 (-0.6, 0.9)	0.3 (0.0, 0.6)	0.1 (-0.1, 0.3)	-0.1 (-0.3, 0.1)

[1] Primary exposure was the normalized (to body mass) value, models were adjusted for sex, baseline age, baseline treatment, limb dominance, and baseline exposure and outcome (KOOS outcome) and sex, baseline age, baseline height, limb dominance, baseline sport type, and baseline exposure and outcome (YBT and hop outcome)

[2] All models were adjusted for baseline sport type, baseline competition level, baseline weight, and baseline outcome and exposure value. Baseline height was adjusted in the Hop and YBT outcome models.

[3] A complete case analysis, following the primary models was performed in the observed data

Abbreviations: cm (centimeter), CI (confidence interval), KOOS (Knee injury and Osteoarthritis Outcome Score), YBT (Y-Balance Test)

*Beta coefficients were back transformed to the response scale (i.e., % KOOS score from 0 to 100)

**(0 represents baseline assessment, 6 represents the 6-month follow-up and 12 represents the 12-month follow-up)
